# Soil carbon and nitrogen data during eight years of cover crop and compost treatments in organic vegetable production

**DOI:** 10.1016/j.dib.2020.106481

**Published:** 2020-11-01

**Authors:** Kathryn E. White, Eric B. Brennan, Michel A. Cavigelli

**Affiliations:** Department of Agriculture, Agricultural Research Service, United States

**Keywords:** Soil carbon, Nitrogen, Cover crops, Compost, Organic vegetable production, Organic farming, Nutrient management, Nitrogen budgets

## Abstract

Data presented are on carbon (C) and nitrogen (N) inputs, and changes in soil C and N in eight systems during the first eight years of a tillage-intensive organic vegetable systems study that was focused on romaine lettuce and broccoli production in Salinas Valley on the central coast region of California. The eight systems differed in organic matter inputs from cover crops and urban yard-waste compost. The cover crops included cereal rye, a legume-rye mixture, and a mustard mixture planted at two seeding rates (standard rate 1x versus high rate 3x). There were three legume-rye 3x systems that differed in compost inputs (0 versus 7.6 Mg ha^−1^ vegetable crop^−1^) and cover cropping frequency (every winter versus every fourth winter). The data include: (1) changes in soil total organic C and total N concentrations and stocks and nitrate N (NO_3_–N) concentrations over 8 years, (2) cumulative above ground and estimated below ground C and N inputs, cover crop and crop N uptake, and harvested crop N export over 8 years, (3) soil permanganate oxidizable carbon (POX-C) concentrations and stocks at time 0, 6 and 8 years, and (4) cumulative, estimated yields of lettuce and broccoli (using total biomass and harvest index values) over the 8 years. The C inputs from the vegetables and cover crops included estimates of below ground inputs based on shoot biomass and literature values for shoot:root. The data in this article support and augment information presented in the research article “Winter cover crops increase readily decomposable soil carbon, but compost drives total soil carbon during eight years of intensive, organic vegetable production in California”.

## Specifications Table

**Table:** 

Subject	*Agriculture*
Specific subject area	Soil carbon and nitrogen, soil carbon sequestration, carbon and nitrogen budgets, nutrient management, vegetable production, long-term organic systems research
Type of data	Table Figure
How data were acquired	Samples of cover crop and vegetable shoots were collected in the field and oven-dried to obtain dry matter. Soil samples were collected in the field and air dried. All samples were analyzed in a laboratory for total carbon and nitrogen using a TruSpec CN analyzer (LECO Corp., Saint Joseph, MI). Soil nitrate concentrations were determined by flow injection photometric analysis of 2.0 N KCl extracts.
Data format	Raw Descriptive Inferential
Parameters for data collection	*Factors that vary among systems are cover cropping frequency, cover crop type, cover crop seeding rate, and compost application rate.*
Description of data collection	*Eight intensive organic vegetable cropping systems were evaluated over an eight year period. Cover crop biomass was sampled in spring prior to incorporation. Vegetables were harvested at maturity by commercial crews. Soils were sampled prior to cover crop planting in fall.*
Data source location	*Salinas, California, United States of America. lat. 36.622658, long. -121.549172, elevation 37m above sea level.*
Data accessibility	With the article
Related research article	White K.E., E.B. Brennan, M.A. Cavigelli, R.F. Smith. 2020. Winter cover crops increase readily decomposable soil carbon, but compost drives total soil carbon during eight years of intensive, organic vegetable production in California. PLoS ONE 15:e0228677.

## Value of the Data

• The data are from the first eight years of the longest running organic systems study in the U.S. that is focused on high-value, high-input, tillage-intensive, organic vegetable production. Salinas, CA is the most important region of the U.S. for high-value, cool season vegetable production.
• The impact of intensively tilled vegetable systems with cover crop and compost inputs on soil C and N stocks is poorly understood. This data could be valuable in future meta-analyses that seek to understand the complex effects of compost and cover crops on soil properties in vegetable systems. The data augment our related publications that only included data from 5 of the 8 systems with cover crop seeding rates that provided optimum weed suppression in the long-term study. The additional systems include the same cover crops at different seeding rates.
• The data may serve as a benchmark for future studies of soil organic C and total N changes in a loamy sand soil in California and other regions with a Mediterranean climate.
• This data may be useful to develop more sustainable organic and conventional vegetable systems in many regions of the world. For example, it may serve as a benchmark in the development of reduced tillage systems and improved nutrient management for vegetable production in this region and elsewhere.
• This data enables others to independently evaluate or extend the statistical analyses presented in the related articles. This may be useful to help researchers and students understand the statistical analysis approach that focused on point and interval estimates in the related articles. This statistical analysis approach used the Exploratory Software for Confidence Intervals (ESCI) software that is freely available online (see link below).

## 1 Data Description

**Fig. 1:**
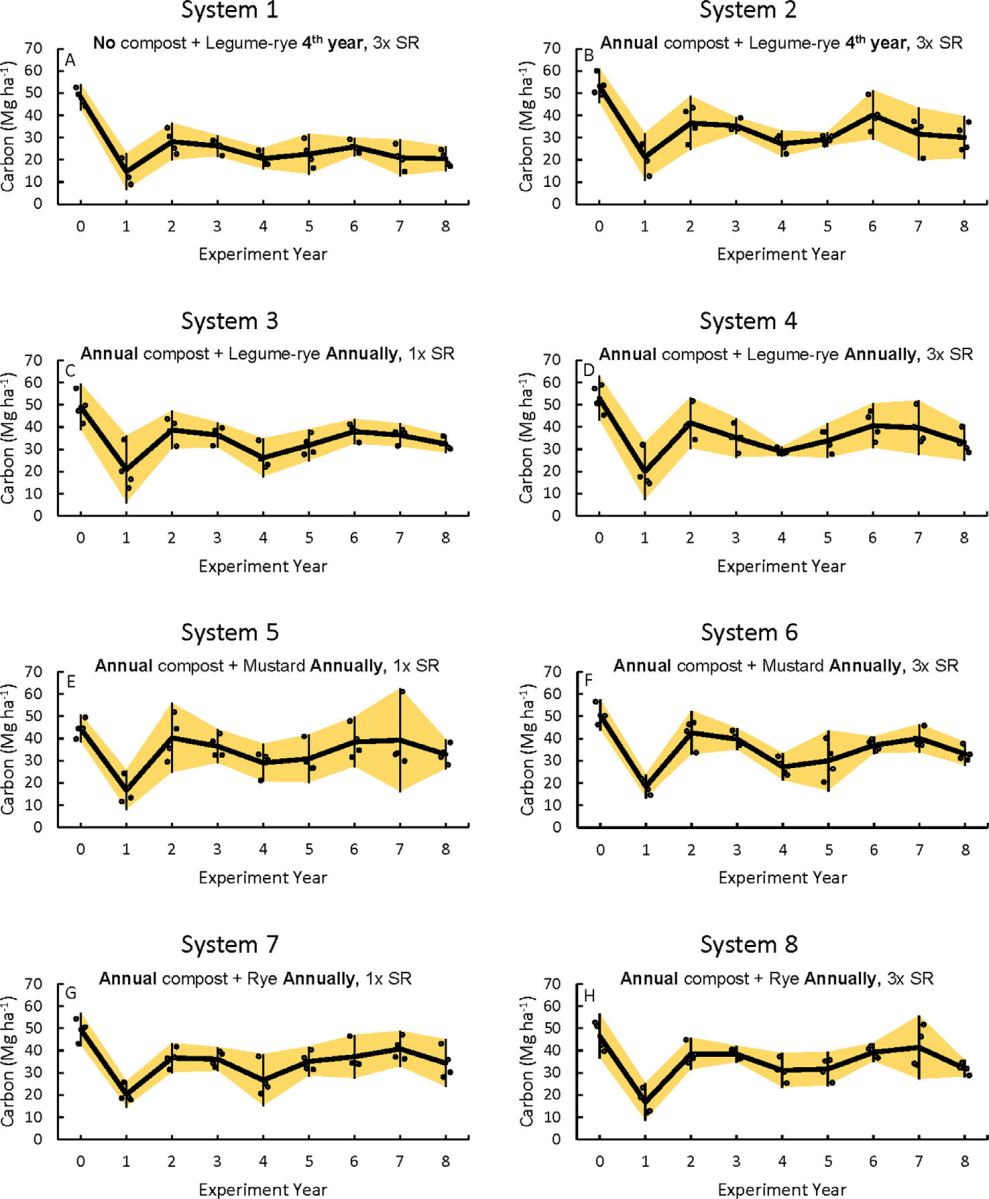
Total organic carbon stocks for the 0 to 30 cm depth in all eight systems (A)–(H) over eight years in the Salinas Organic Cropping Systems experiment in Salinas, California. The systems differed in compost additions (none versus 7.6 Mg ha^−1^ before each vegetable crop, oven-dry basis), cover crop type (legume-rye, mustard, or rye), cover cropping frequency (every 4th winter versus annually) and cover crop seeding rate (1x= standard rate versus 3x= high rate); see Table 1 for more seeding rate details. Symbols are raw data in order of replicates 1 to 4 with mean and 95% confidence interval (CI) in the center of each data cluster.

**Fig. 2:**
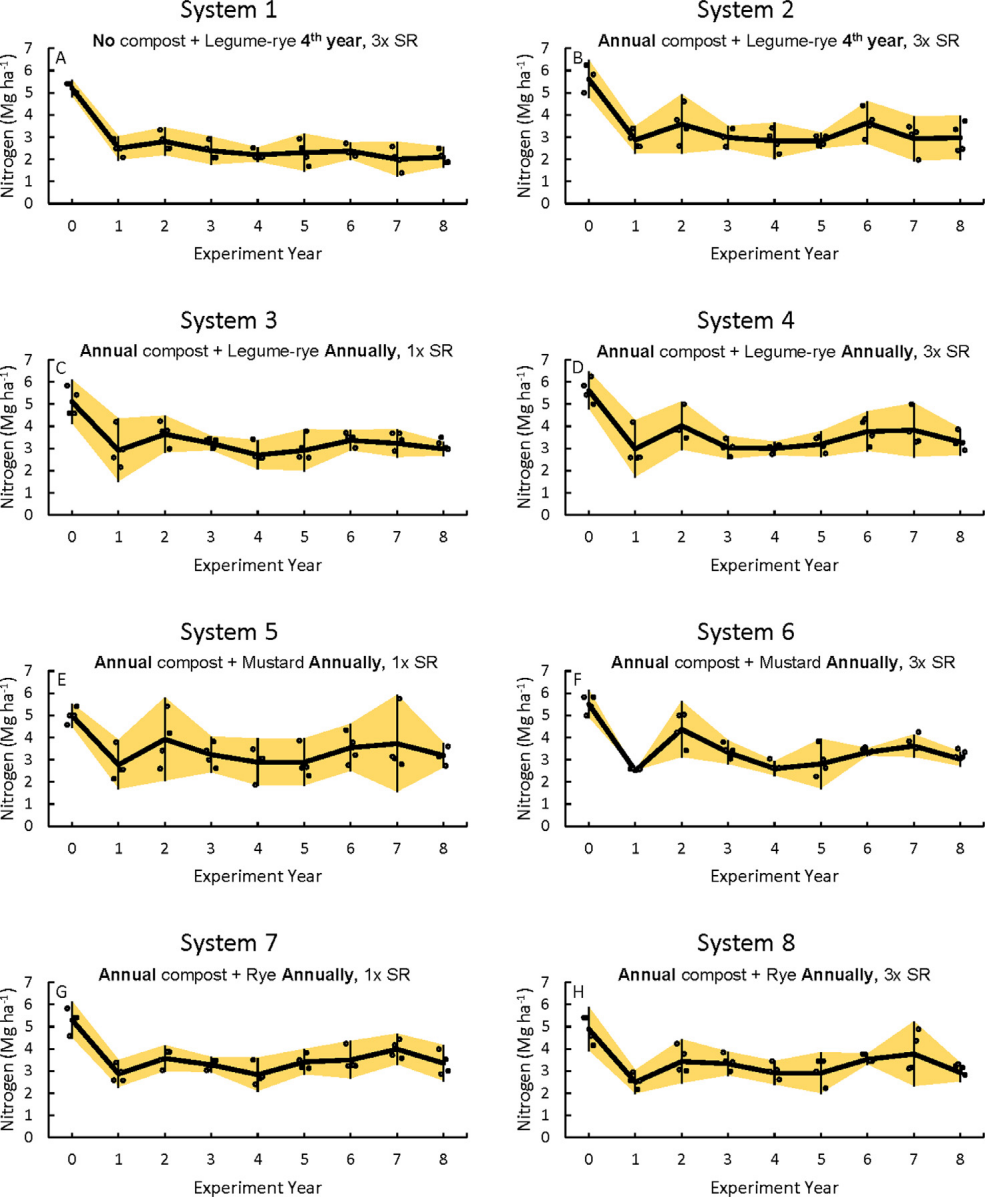
Total nitrogen stocks for the 0 to 30 cm depth in all eight systems (A)–(H) over eight years in the Salinas Organic Cropping Systems experiment in Salinas, California. The systems differed in compost additions (none versus 7.6Mg ha^−1^ before each vegetable crop, oven-dry basis), cover crop type (legume-rye, mustard, or rye), cover cropping frequency (every 4th winter versus annually) and cover crop seeding rate (1x= standard rate versus 3x= high rate); see Table 1 for more seeding rate details. Symbols are raw data in order of replicates 1 to 4 with mean and 95% confidence interval (CI) in the center of each data cluster.

**Fig. 3:**
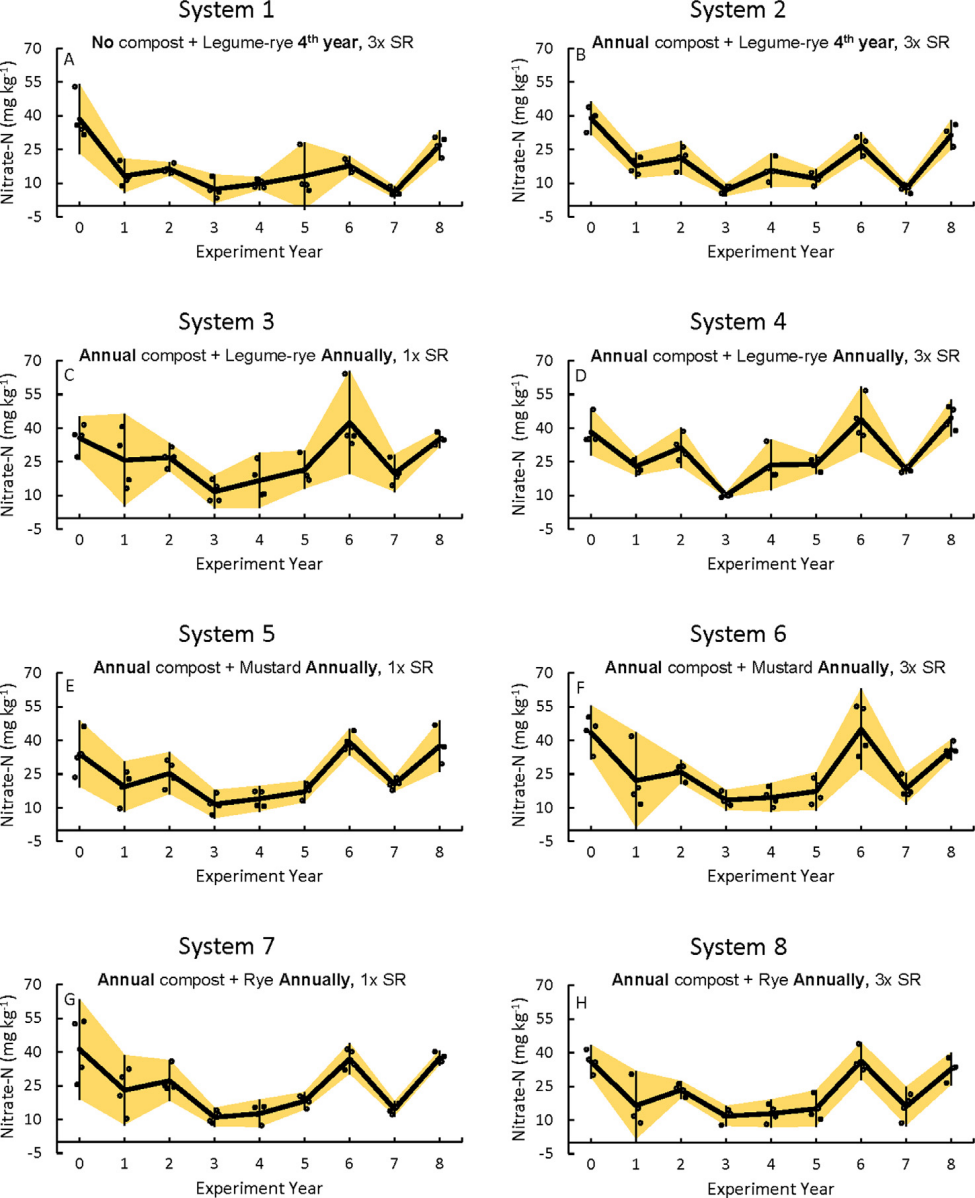
Nitrate nitrogen concentrations for the 0 to 30 cm depth prior to cover crop planting in all eight systems (A-H) over eight years in the Salinas Organic Cropping Systems experiment in Salinas, California. The systems differed in compost additions (none versus 7.6 Mg ha^−1^ before each vegetable crop, oven-dry basis), cover crop type (legume-rye, mustard, or rye), cover cropping frequency (every 4th winter versus annually) and cover crop seeding rate (1x= standard rate versus 3x= high rate); see Table 1 for more seeding rate details. Symbols are raw data in order of replicates 1 to 4 with mean and 95% confidence interval (CI) in the center of each data cluster.

**Fig. 4:**
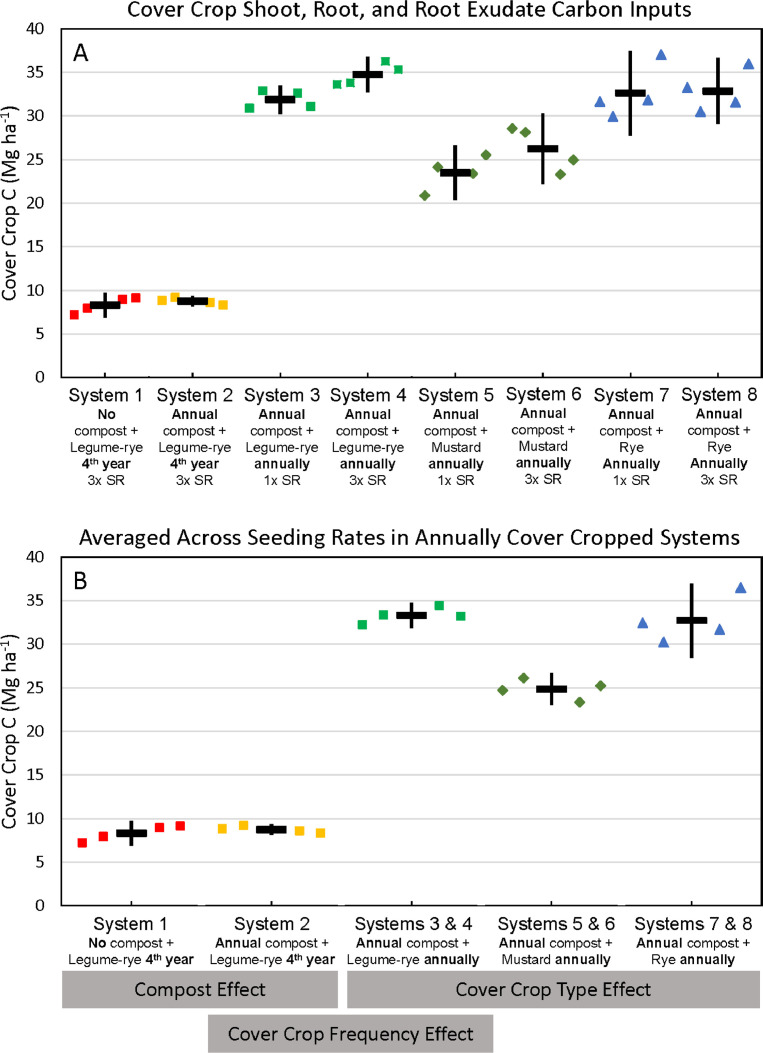
Cumulative carbon inputs from cover crop shoots, roots and root exudates in all eight systems (A) and averaged across the 1x and 3x seeding rates (SR) in the annually cover cropped systems (B) following 8 years of the Salinas Organic Cropping Systems experiment in Salinas, California. The systems differed in compost additions (none versus 7.6 Mg ha^−1^ before each vegetable crop, oven-dry basis), cover crop type (legume-rye, mustard, or rye), cover cropping frequency (every 4th winter versus annually) and cover crop seeding rate (1x= standard rate versus 3x= high rate); see Table 1 for more seeding rate details. Symbols are raw data in order of replicates 1 to 4 with mean and 95% confidence interval (CI) in the center of each data cluster. The rectangular boxes below the system labels on the *x*-axis in plot B show the systems that can be compared to evaluate the effects of compost, cover crop frequency, and cover crop type.

**Fig. 5:**
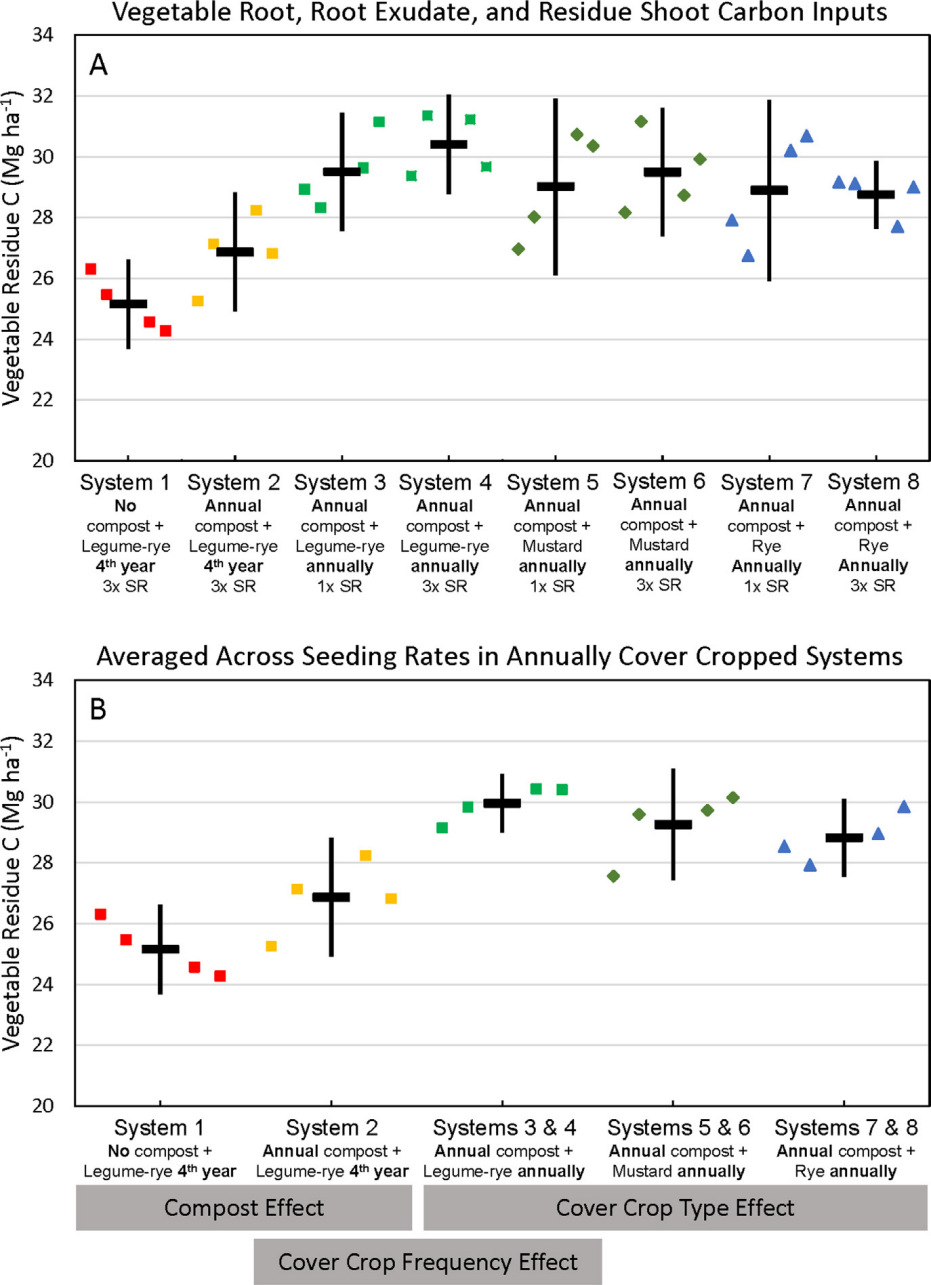
Cumulative carbon inputs from vegetable roots, root exudates and shoot residues in all eight systems (A) and averaged across the 1x and 3x seeding rates (SR) in the annually cover cropped systems (B) following 8 years of the Salinas Organic Cropping Systems experiment in Salinas, California. The systems differed in compost additions (none versus 7.6 Mg ha^−1^, before each vegetable crop, oven-dry basis) cover crop type (legume-rye, mustard, or rye), cover cropping frequency (every 4th winter versus annually) and cover crop seeding rate (1x= standard rate versus 3x= high rate); see Table 1 for more seeding rate details. Symbols are raw data in order of replicates 1 to 4 with mean and 95% confidence interval (CI) in the center of each data cluster. The rectangular boxes below the system labels on the *x*-axis in plot B show the systems that can be compared to evaluate the effects of compost, cover crop frequency, and cover crop type.

**Fig. 6:**
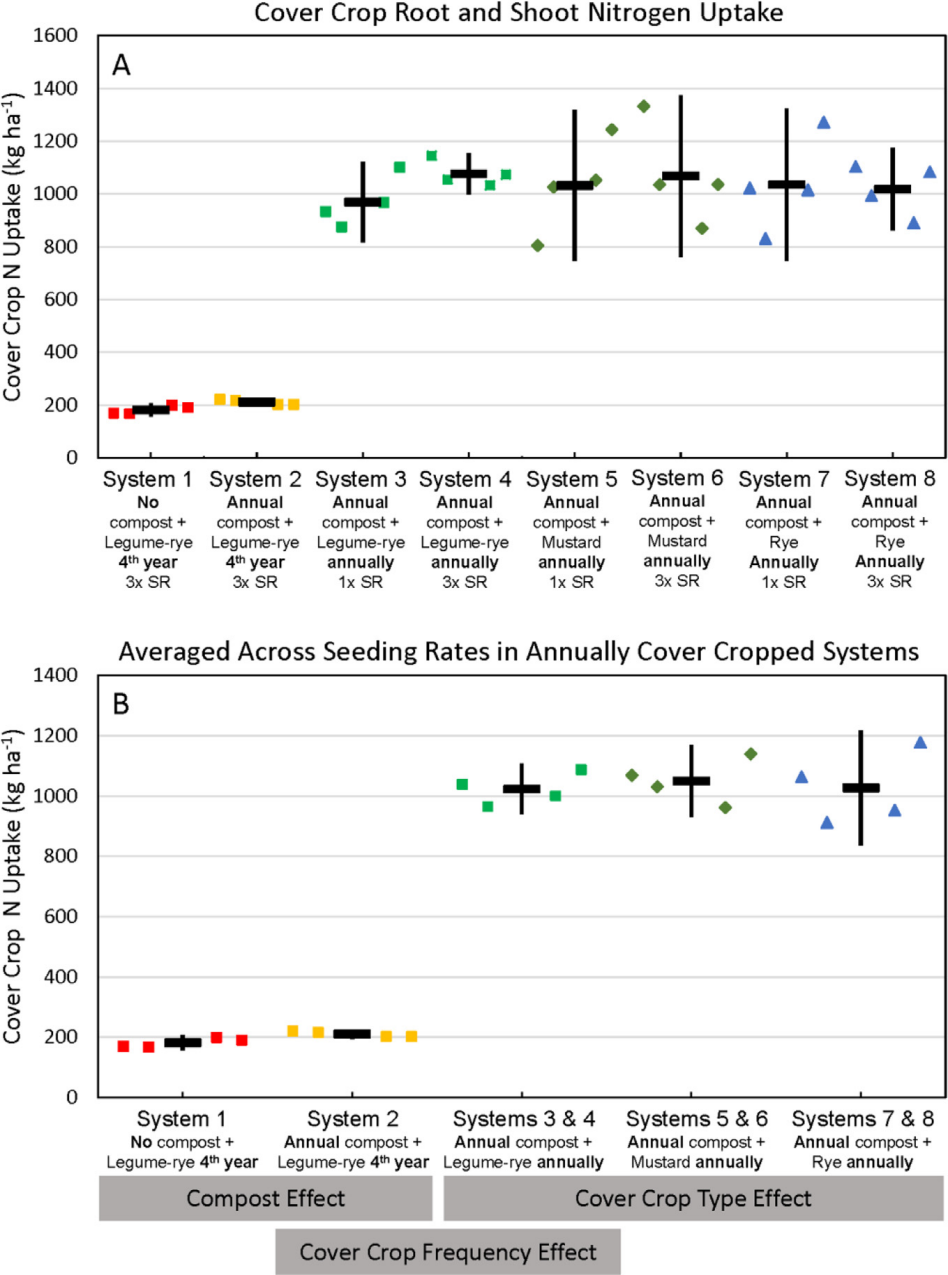
Cumulative nitrogen uptake by cover crop shoots and roots in all eight systems (A) and averaged across the 1x and 3x seeding rates (SR) in the annually cover cropped systems (B) following 8 years of the Salinas Organic Cropping Systems experiment in Salinas, California. Nitrogen uptake in the legume-rye systems does not include legume nitrogen fixation. Nitrogen uptake by roots is based on estimated root biomass and assuming a 20% lower N concentration in roots compared to shoots [9]. The systems differed in compost additions (none versus 7.6 Mg ha^−1^ before each vegetable crop, oven-dry basis), cover crop type (legume-rye, mustard, or rye), cover cropping frequency (every 4th winter versus annually) and cover crop seeding rate (1x= standard rate versus 3x= high rate); see Table 1 for more seeding rate details. Symbols are raw data in order of replicates 1 to 4 with mean and 95% confidence interval (CI) in the center of each data cluster. The rectangular boxes below the system labels on the *x*-axis in plot B show the systems that can be compared to evaluate the effects of compost, cover crop frequency, and cover crop type.

**Fig. 7:**
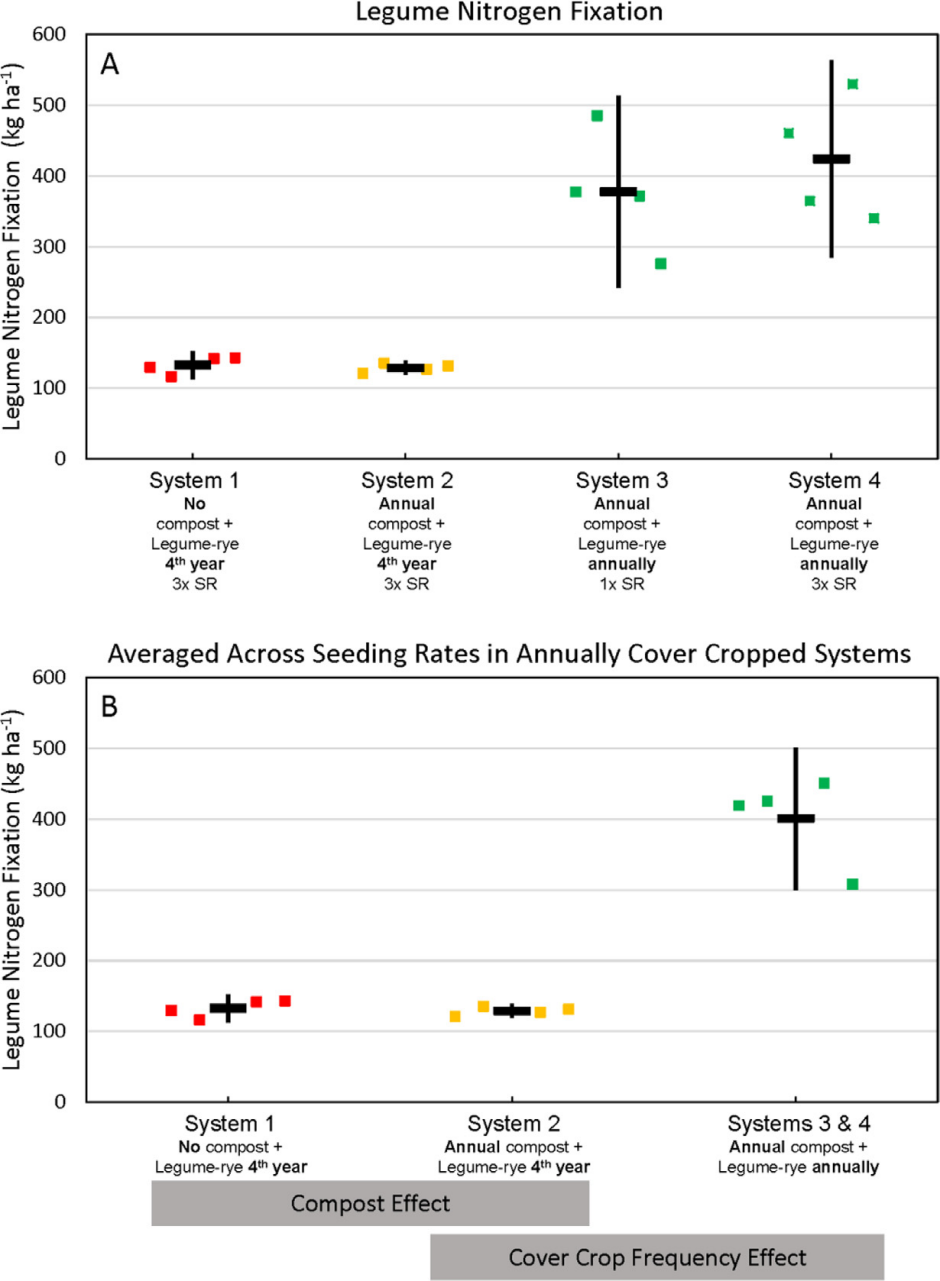
Cumulative, estimated nitrogen fixation by legumes in all four systems with legume-rye cover crops (A) and averaged across the 1x and 3x seeding rates (SR) in the annually cover cropped systems (B) during eight years of the Salinas Organic Cropping Systems experiment in Salinas, California. Nitrogen in roots is based on estimated root biomass and assuming a 20% lower N concentration in roots compared to shoots [9]. The systems differed in compost additions (none versus 7.6 Mg ha^−1^ before each vegetable crop, oven-dry basis), cover cropping frequency (every 4th winter versus annually) and cover crop seeding rate (1x= standard rate versus 3x= high rate); see Table 1 for more seeding rate details. Symbols are raw data in order of replicates 1 to 4 with mean and 95% confidence interval (CI) in the center of each data cluster. The rectangular boxes below the system labels on the *x*-axis in plot B show the systems that can be compared to evaluate the effects of compost and cover crop frequency.

**Fig. 8:**
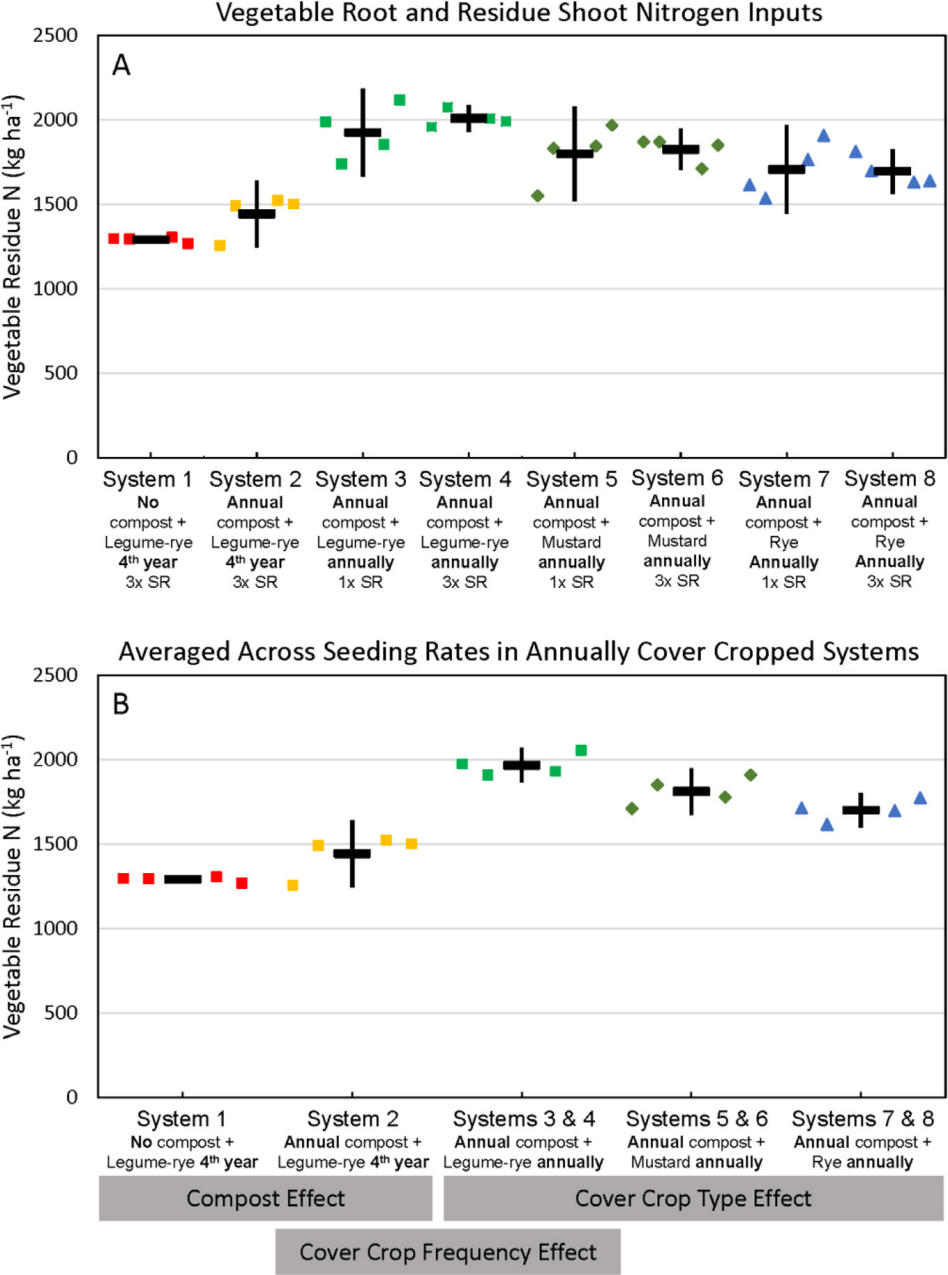
Cumulative nitrogen inputs returned to the soil from vegetable roots and residue shoots following harvest in all eight systems (A) and averaged across the 1x and 3x seeding rates (SR) in the annually cover cropped systems (B) following 8 years of the Salinas Organic Cropping Systems experiment in Salinas, California. Nitrogen input by roots is based on estimated root biomass and assuming a 20% lower N concentration in roots compared to shoots [9]. The systems differed in compost additions (none versus 7.6 Mg ha^−1^ 1 before each vegetable crop, oven-dry basis), cover crop type (legume-rye, mustard, or rye), cover cropping frequency (every 4th winter versus annually) and cover crop seeding rate (1x= standard rate versus 3x= high rate); see Table 1 for more seeding rate details. Symbols are raw data in order of replicates 1 to 4 with mean and 95% confidence interval (CI) in the center of each data cluster. The rectangular boxes below the system labels on the *x*-axis in plot B show the systems that can be compared to evaluate the effects of compost, cover crop frequency, and cover crop type.

**Fig. 9:**
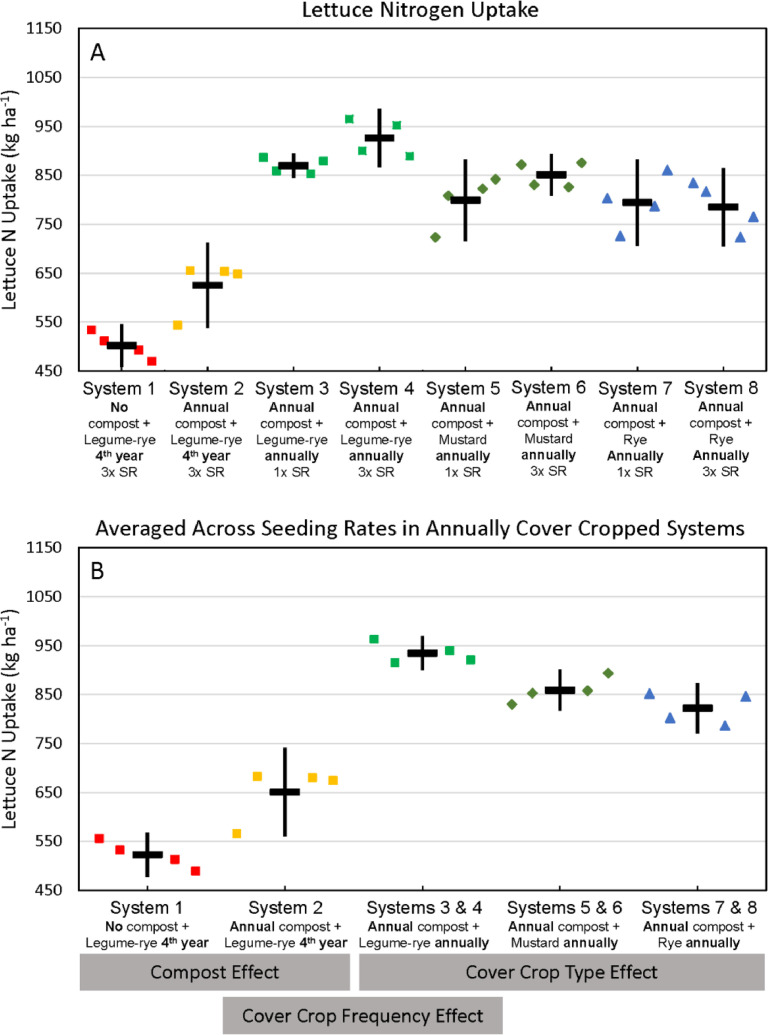
Cumulative nitrogen uptake by lettuce shoots and roots in all eight systems (A) and averaged across the 1x and 3x seeding rates (SR) in the annually cover cropped systems (B) following 8 years of the Salinas Organic Cropping Systems experiment in Salinas, California. Nitrogen uptake by roots is based on estimated root biomass and assuming a 20% lower N concentration in roots compared to shoots [9]. The systems differed in compost additions (none versus 7.6 Mg ha^−1^ before each vegetable crop, oven-dry basis), cover crop type (legume-rye, mustard, or rye), cover cropping frequency (every 4th winter versus annually) and cover crop seeding rate (1x= standard rate versus 3x= high rate); see Table 1 for more seeding rate details Symbols are raw data in order of replicates 1 to 4 with mean and 95% confidence interval (CI) in the center of each data cluster. The rectangular boxes below the system labels on the *x*-axis in plot B show the systems that can be compared to evaluate the effects of compost, cover crop frequency, and cover crop type.

**Fig. 10:**
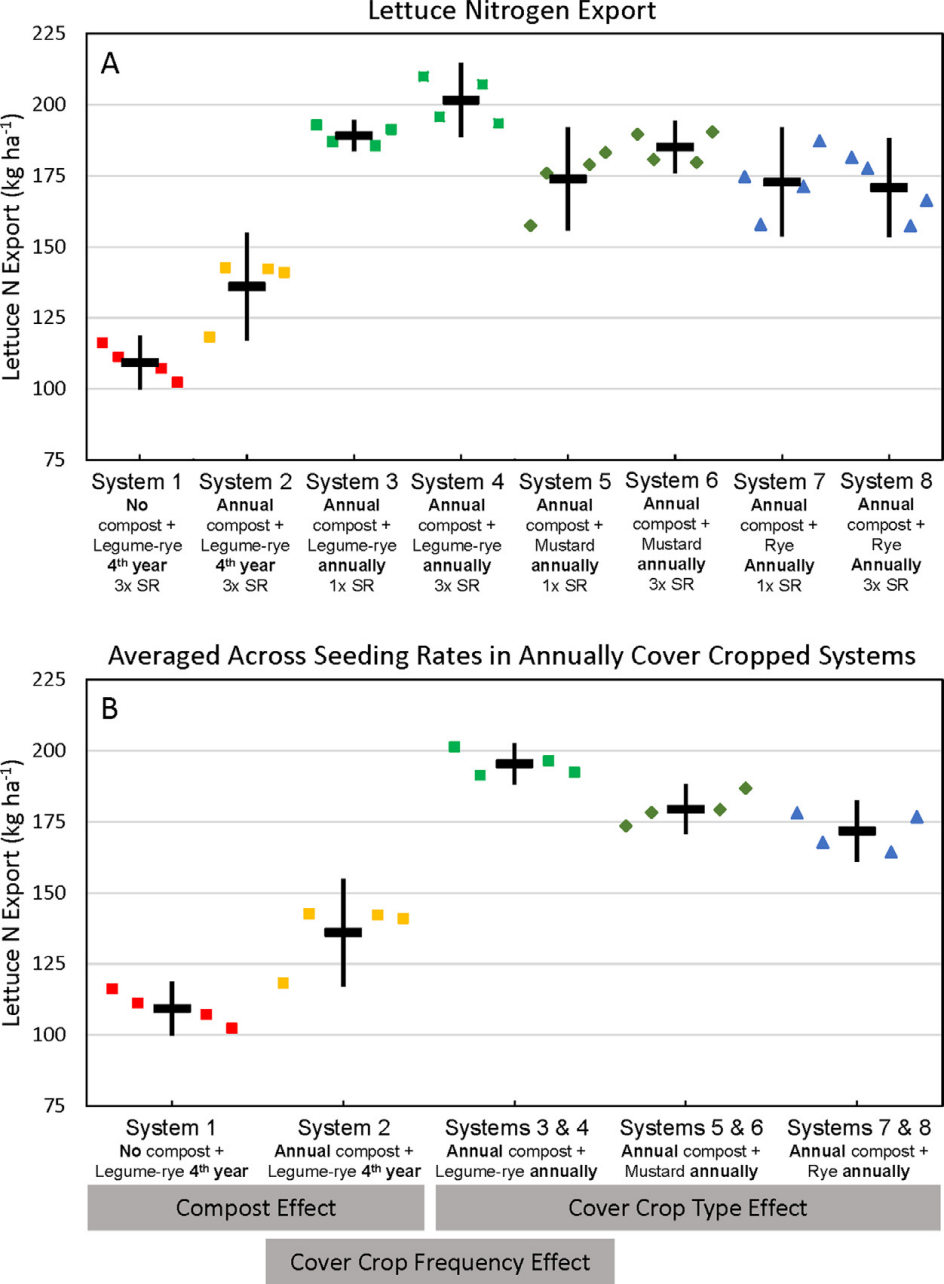
Cumulative nitrogen export in lettuce harvest in all eight systems (A) and averaged across the 1x and 3x seeding rates (SR) in the annually cover cropped systems (B) following 8 years of the Salinas Organic Cropping Systems experiment in Salinas, California. The systems differed in compost additions (none versus 7.6 Mg ha^−1^ before each vegetable crop, oven-dry basis), cover crop type (legume-rye, mustard, or rye), cover cropping frequency (every 4th winter versus annually) and cover crop seeding rate (1x= standard rate versus 3x= high rate); see Table 1 for more seeding rate details. Symbols are raw data in order of replicates 1 to 4 with mean and 95% confidence interval (CI) in the center of each data cluster. The rectangular boxes below the system labels on the *x*-axis in plot B show the systems that can be compared to evaluate the effects of compost, cover crop frequency, and cover crop type.

**Fig. 11:**
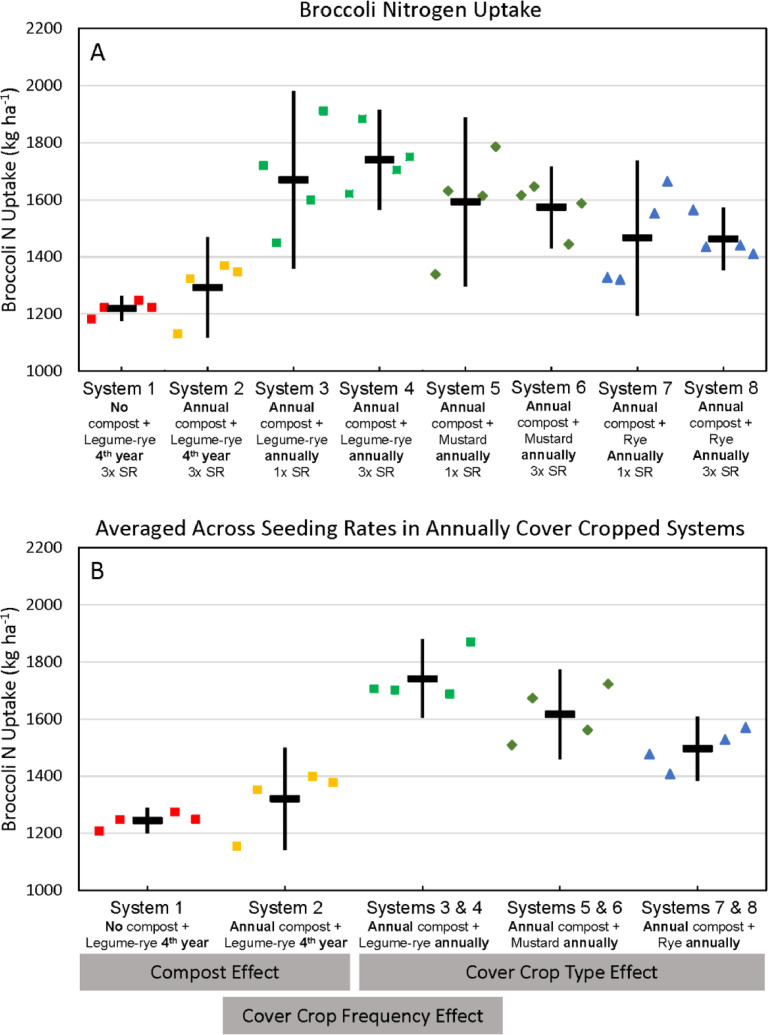
Cumulative nitrogen uptake by broccoli shoots and roots in all eight systems (A) and averaged across the 1x and 3x seeding rates (SR) in the annually cover cropped systems (B) following 8 years of the Salinas Organic Cropping Systems experiment in Salinas, California. Nitrogen uptake by roots is based on estimated root biomass and assuming a 20% lower N concentration in roots compared to shoots [9]. The systems differed in compost additions (none versus 7.6 Mg ha^−1^ before each vegetable crop, oven-dry basis), cover crop type (legume-rye, mustard, or rye), cover cropping frequency (every 4th winter versus annually) and cover crop seeding rate (1x= standard rate versus 3x= high rate); see Table 1 for more seeding rate details. Symbols are raw data in order of replicates 1 to 4 with mean and 95% confidence interval (CI) in the center of each data cluster. The rectangular boxes below the system labels on the *x*-axis in plot B show the systems that can be compared to evaluate the effects of compost, cover crop frequency, and cover crop type.

**Fig. 12:**
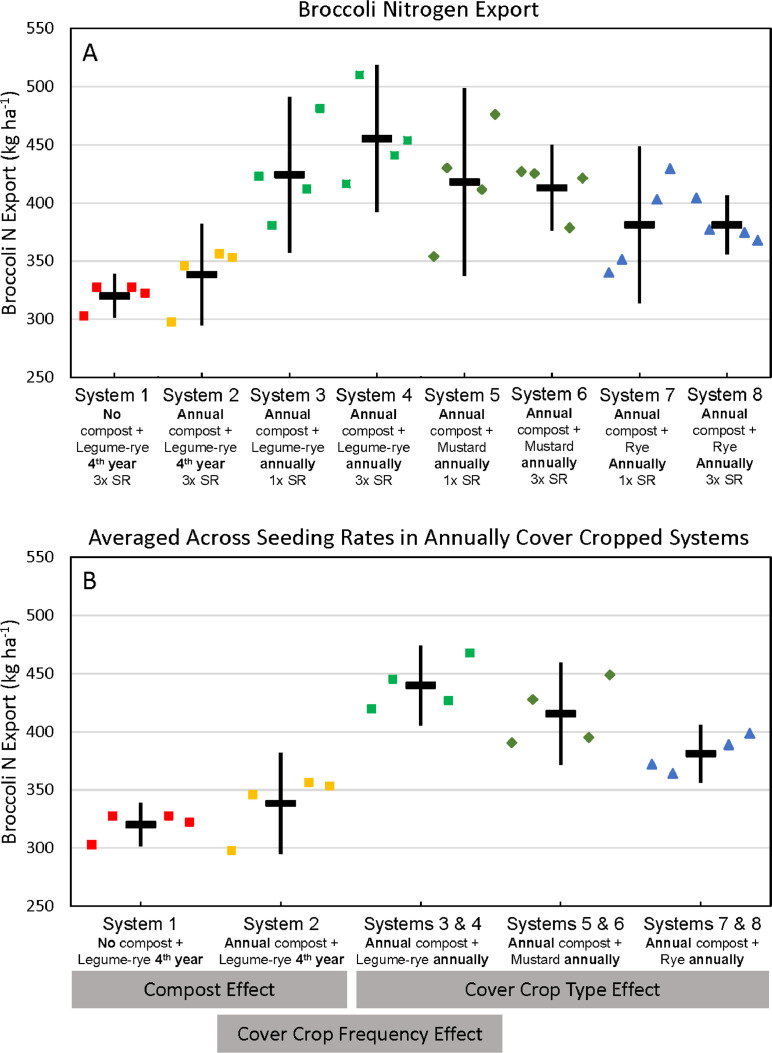
Cumulative nitrogen export in broccoli harvest in all eight systems (A) and averaged across the 1x and 3x seeding rates (SR) in the annually cover cropped systems (B) following 8 years of the Salinas Organic Cropping Systems experiment in Salinas, California. The systems differed in compost additions (none versus 7.6 Mg ha^−1^ before each vegetable crop, oven-dry basis), cover crop type (legume-rye, mustard, or rye), cover cropping frequency (every 4th winter versus annually) and cover crop seeding rate (1x= standard rate versus 3x= high rate); see Table 1 for more seeding rate details. Symbols are raw data in order of replicates 1 to 4 with mean and 95% confidence interval (CI) in the center of each data cluster. The rectangular boxes below the system labels on the *x*-axis in plot B show the systems that can be compared to evaluate the effects of compost, cover crop frequency, and cover crop type.

**Fig. 13:**
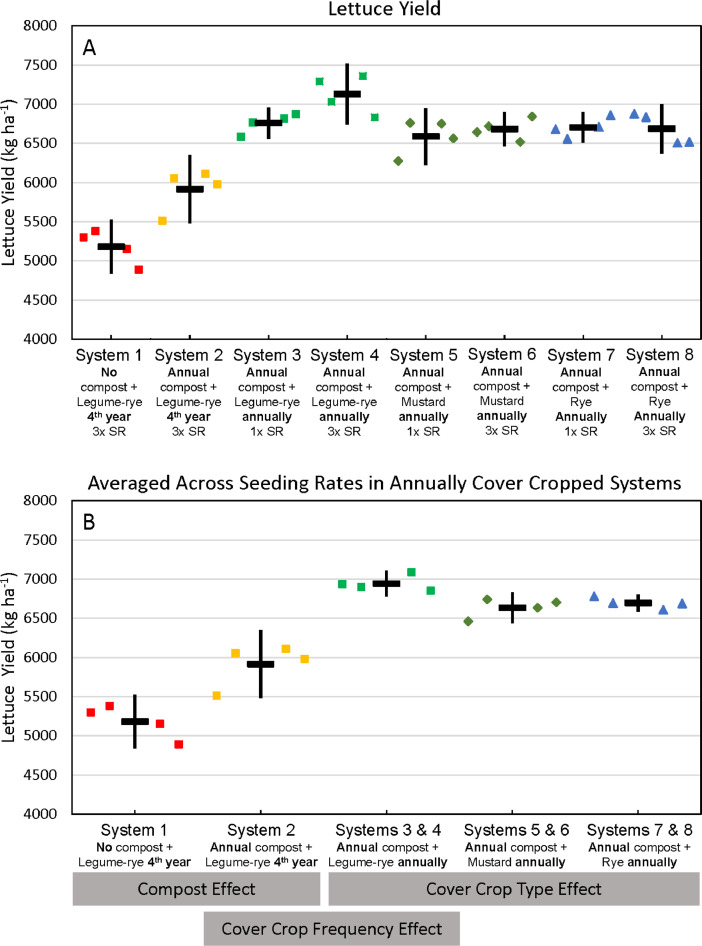
Cumulative lettuce yields in all eight systems (A) and averaged across the 1x and 3x seeding rates (SR) in the annually cover cropped systems (B) following 8 years of the Salinas Organic Cropping Systems experiment in Salinas, California; yields are on an oven-dry basis. The systems differed in compost additions (none versus 7.6 Mg ha^−1^ before each vegetable crop, oven-dry basis), cover crop type (legume-rye, mustard, or rye), cover cropping frequency (every 4th winter versus annually) and cover crop seeding rate (1x= standard rate versus 3x= high rate); see Table 1 for more seeding rate details. Symbols are raw data in order of replicates 1 to 4 with mean and 95% confidence interval (CI) in the center of each data cluster. The rectangular boxes below the system labels on the *x*-axis in plot B show the systems that can be compared to evaluate the effects of compost, cover crop frequency, and cover crop type.

**Fig. 14:**
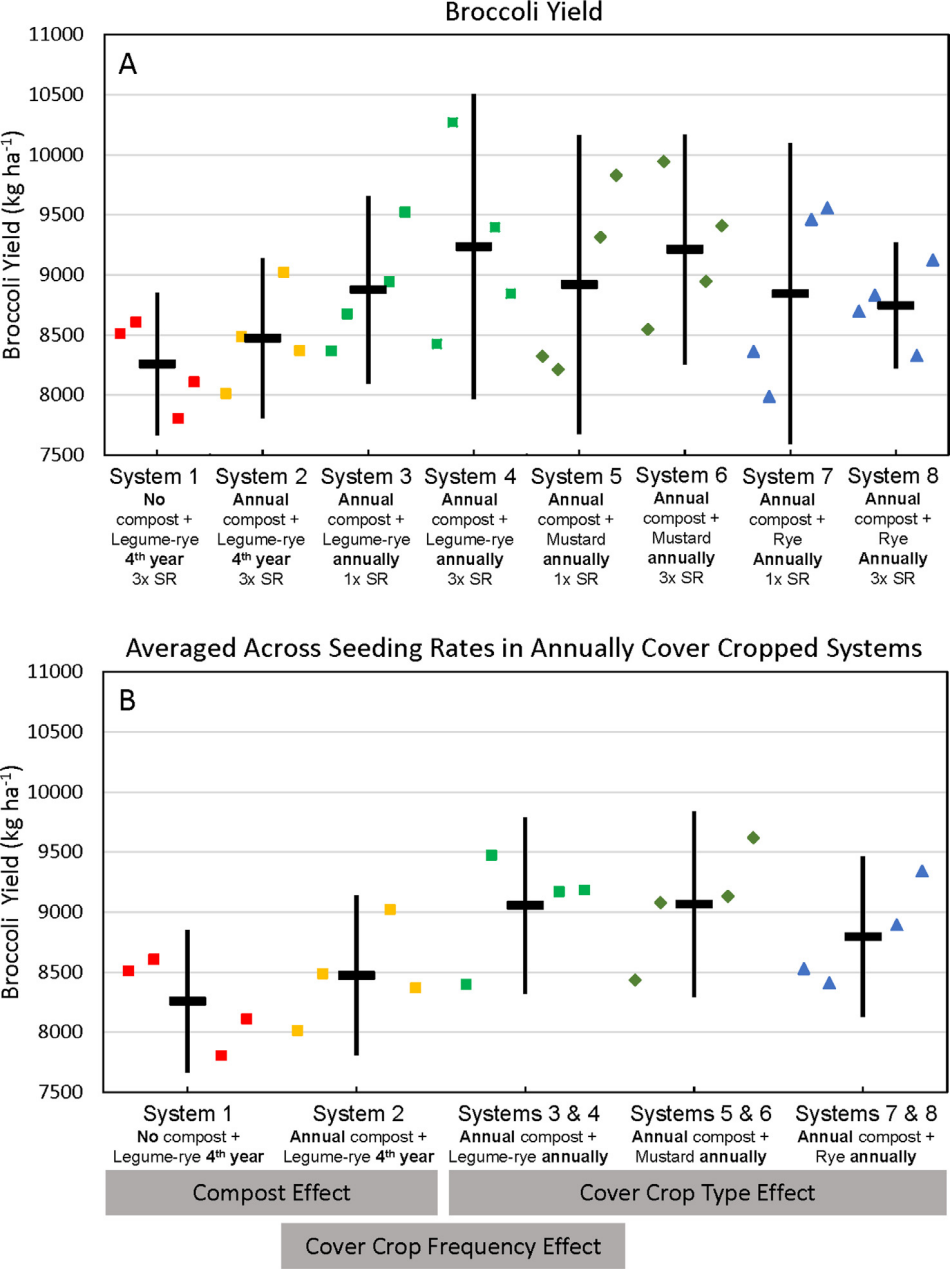
Cumulative broccoli yields in all eight systems (A) and averaged across the 1x and 3x seeding rates (SR) in the annually cover cropped systems (B) following 8 years of the Salinas Organic Cropping Systems experiment in Salinas, California; yields are on an oven-dry basis. The systems differed in compost additions (none versus 7.6 Mg ha^−1^ before each vegetable crop, oven-dry basis), cover crop type (legume-rye, mustard, or rye), cover cropping frequency (every 4th winter versus annually) and cover crop seeding rate (1x= standard rate versus 3x= high rate); see Table 1 for more seeding rate details. Symbols are raw data in order of replicates 1 to 4 with mean and 95% confidence interval (CI) in the center of each data cluster. The rectangular boxes below the system labels on the *x*-axis in plot B show the systems that can be compared to evaluate the effects of compost, cover crop frequency, and cover crop type.

This article includes the raw data, descriptive data (means) and inferential statistics (95% confidence intervals) on the effects of compost and cover cropping over an 8 year period in the Salinas Organic Cropping Systems (SOCS) experiment including: (1) changes in soil total organic carbon (C) and total nitrogen (N) concentrations and stocks and nitrate N (NO_3_-N) concentrations over 8 years (Table 2, Figs 1–3), (2) cumulative above and estimated below ground C and N inputs, cover crop and crop N uptake, and harvested crop N export (Table 3, Figs 4–12), (3) soil permanganate oxidizable carbon (POX-C) concentrations, stocks and changes in POX-C between the beginning of the study and after 6 and 8 years (Table 4), and (4) cumulative, estimated yields of lettuce and broccoli over the eight years (Table 4, Fig. 13, Fig. 14) that were removed from the field by commercial crews. Table 2, Table 3, Table 4, Table 5 are available in a spreadsheet in the supplementary material (Supplemental Tables 1–4). Yields are estimated based on measured crop biomass and typical harvest indices. This important long-term study is located at the USDA-ARS (United States Department of Agriculture – Agricultural Research Service) organic research farm in Salinas, California and is approximately 24 km inland from Monterey Bay in a region commonly referred to as the ‘Salad Bowl of America’. This ongoing systems study was designed to provide information on the impact of urban yard waste compost and cover crops (type, frequency, and seeding rate) on a variety measures of sustainability (ex., soil health, yields, weeds) of vegetable production.

## 2 Experimental Design, Materials and Methods

The ongoing SOCS experiment began in 2003 and is located in a 0.9 ha field that includes 32 plots, organized in 4 blocks of 8 systems plots per block. The first eight years of this study were focused on vegetable production (lettuce followed by broccoli most years) in 8 systems that differed in compost inputs and cover crop (type, seeding rate and frequency) (Table 1). The annual rotation began in October or November each year and included either a winter fallow or winter cover crop that grew until February or March and was usually followed by the two vegetable crops. Winter weed growth in systems 1 and 2 that were fallow most winters were managed with shallow tillage as needed, to minimize weed growth and prevent weed seed production; otherwise, tillage was consistent across all systems. Other than the differences in cover crop and compost inputs among systems, all management (i.e. pest control, tillage, harvest schedules) and inputs (i.e. irrigation, fertilizers) were equivalent across all systems for the vegetable crops [1], [2], [3], [4].

**Table 1:** Descriptions of systems in the Salinas Organic Cropping Systems experiment in Salinas, California.

		–––-Cover crop–––-			

System ID used in this *Data in Brief* article	System ID in PLoS One article^1^	Type^2^	Frequency^3^	Seeding rate^4^	Compost input^5^
1*	1	Legume-rye	4th Winter	3x	No
2*	2	Legume-rye	4th Winter	3x	Yes
3*		Legume-rye	Every Winter	1x	Yes
4*	3	Legume-rye	Every Winter	3x	Yes
5*	4	Mustard	Every Winter	1x	Yes
6*		Mustard	Every Winter	3x	Yes
7*	5	Rye	Every Winter	1x	Yes
8*		Rye	Every Winter	3x	Yes

1 System ID code used in the related article [1].

2 By seed weight, the legume-rye mixture included 10% Rye (‘Merced’ *Secale cereale* L.), 35% Faba bean, (*Vicia faba* L.; small-seeded type known as ‘bell bean’), 25% Pea, ‘Magnus’ *Pisum sativum* L., 15% common vetch, *V. sativa* L., and 15% purple vetch, *V. benghalensis* L. By seed weight mustard included 61% white mustard, ‘IdaGold’ *Sinapis alba* L., and 39% India mustard, ‘Pacific Gold’ *Brassica juncea* Czern.

3 Systems 1 and 2 were fallow all winters except the winter of year 4 and 8. All other systems were cover cropped every winter.

4 The 1x and 3x rates in kg ha^−1^ were 11 and 33 for mustard (61% ‘Ida Gold’ white mustard (Sinapis alba L.), 39% ‘Pacific Gold’ Indian mustard (Brassica juncea Czern.) by seed weight), 90 and 270 for rye (‘Merced’ rye (Secale cereale L.), and 140 and 420 for the legume-rye mixture (10% ‘Merced’ rye, 35% faba bean, 25% ‘Magnus’ pea, 15% common vetch and 15% purple vetch by seed weight).

5 The compost was made from urban yard waste and the application rate (oven dry basis) prior to each vegetable crop was 7.6 Mg ha^−1^. Two vegetable crops were grown annually in all years except year 8 when only one vegetable was grown.

Cover crop shoot C and N inputs were calculated based on previously published shoot biomass [2] and C concentration [5] data collected just prior to termination from this study. The vegetable post-harvest residues were estimated based on mature lettuce and broccoli oven-dry shoot biomass assuming harvest indices of 0.26 and 0.24, respectively. To estimate the N exported from the field in the harvested vegetables we multiplied the total shoot N content by the harvest index for lettuce, whereas for broccoli the total shoot N content was multiplied by 0.31 based on Smith et al. [6]. Lettuce and broccoli biomass were calculated based on 32 and 20 plants, respectively, harvested from each plot. We estimated below ground C inputs from cover crop and vegetable roots and root exudates based on above ground biomass as described in detail in White et al. [1].

Soil C and N data were measured in a composite soil sample of 20 subsamples collected from the 0 to 30 cm depth in each plot prior to cover crop planting or winter fallow each year. Total soil C and N were determined on all air-dried ground (<0.5 mm) soil samples by combustion and inorganic soil C by titration of carbonate and bicarbonate. Soil organic C was calculated as the difference between total and inorganic soil C. Soil NO_3_-N was measured on air-dried ground (<0.5 mm) soil samples by flow injection photometric analysis of 2.0 N KCl extracts. Soil bulk density was used to convert soil organic C and total N concentrations to stocks (kg ha^−1^) [1,7].

The POX-C analysis was conducted on soil samples collected to a depth of 0 to 6.5 cm from 6 to 8 core samples per plot from time zero and after 6 years that were frozen (-25 C) until analysis. POX-C analysis for year 8 was conducted on air-dried soil collected from the 0 to 30 cm depth. Permanganate oxidizable C was determined using spectrophotometry as described in [1], and converted to POX-C stock using soil bulk density.

The data presented here include the raw data for all eight systems in the experiment (Table 2), whereas the data for only five systems were used in the analyses in the related articles [1,4,8]. Figs 1–14 illustrate major data patterns with the raw data plotted with means and 95% confidence intervals. We refer readers to our recent related article [8] for an explanation of how to compare systems using 95% confidence intervals in this study and how the ESCI software (available at https://thenewstatistics.com/itns/esci/) can help with these comparisons.

**Table 2:** Raw data of soil total organic carbon concentrations, total nitrogen concentrations, nitrate nitrogen concentrations, total organic carbon stocks, and total nitrogen stocks over 8 years from the Salinas Organic Cropping Systems experiment in Salinas, California. This includes data from all eight systems in the experiment. The related article in PLoS ONE [1] only included data from five of the eight systems with optimal seeding rates for weed suppression. A Microsoft Excel version of the table is available in the supplementary material (Supplementary Table 1).

Overview of the data^1^									Soil Carbon and Nitrogen Concentrations			Soil Carbon and Nitrogen Stocks^2^	
Block (i.e. replicate)	Year	Symbol color & shape in PloS One article figures^3^	System ID in *Data in Brief* article^4^	System ID & description used in associated article in *PLoS ONE*^5^	Compost added^6^	Winer cover cropping frequency^7^	Cover crop type^8^	Cover crop seeding rate^8^	Total Organic C	Total N	Nitrate N	Total Organic C	Total N
									mg kg^−1^ soil			Mg ha^−1^	
1	0		1*	1-No Compost + Legume-rye 4th Year	No	Every 4th winter	Leg-rye	3x	12,615	1300	53	52	5.4
2	0		1*	1-No Compost + Legume-rye 4th Year	No	Every 4th winter	Leg-rye	3x	11,883	1300	36	49	5.4
3	0		1*	1-No Compost + Legume-rye 4th Year	No	Every 4th winter	Leg-rye	3x	11,083	1200	34	46	5.0
4	0		1*	1-No Compost + Legume-rye 4th Year	No	Every 4th winter	Leg-rye	3x	10,622	1200	32	44	5.0
1	0		2*	2-Compost + Legume-rye 4th Year	Yes	Every 4th winter	Leg-rye	3x	12,083	1200	33	50	5.0
2	0		2*	2-Compost + Legume-rye 4th Year	Yes	Every 4th winter	Leg-rye	3x	14,430	1500	44	60	6.2
3	0		2*	2-Compost + Legume-rye 4th Year	Yes	Every 4th winter	Leg-rye	3x	11,849	1300	39	49	5.4
4	0		2*	2-Compost + Legume-rye 4th Year	Yes	Every 4th winter	Leg-rye	3x	12,867	1400	40	53	5.8
1	0	NA	3*	NA	Yes	Every winter	Leg-rye	1x	13,815	1400	37	57	5.8
2	0	NA	3*	NA	Yes	Every winter	Leg-rye	1x	11,367	1100	27	47	4.6
3	0	NA	3*	NA	Yes	Every winter	Leg-rye	1x	10,000	1100	37	42	4.6
4	0	NA	3*	NA	Yes	Every winter	Leg-rye	1x	11,953	1300	41	50	5.4
1	0		4*	3-Compost + Legume-rye annually	Yes	Every winter	Leg-rye	3x	13,760	1400	35	57	5.8
2	0		4*	3-Compost + Legume-rye annually	Yes	Every winter	Leg-rye	3x	12,147	1300	35	50	5.4
3	0		4*	3-Compost + Legume-rye annually	Yes	Every winter	Leg-rye	3x	14,151	1500	48	59	6.2
4	0		4*	3-Compost + Legume-rye annually	Yes	Every winter	Leg-rye	3x	10,874	1200	35	45	5.0
1	0		5*	4-Compost + Mustard annually	Yes	Every winter	Mustard	1x	9547	1100	24	40	4.6
2	0		5*	4-Compost + Mustard annually	Yes	Every winter	Mustard	1x	10,660	1200	32	44	5.0
3	0		5*	4-Compost + Mustard annually	Yes	Every winter	Mustard	1x	10,700	1200	34	44	5.0
4	0		5*	4-Compost + Mustard annually	Yes	Every winter	Mustard	1x	11,888	1300	46	49	5.4
1	0	NA	6*	NA	Yes	Every winter	Mustard	3x	13,588	1400	44	56	5.8
2	0	NA	6*	NA	Yes	Every winter	Mustard	3x	11,115	1200	50	46	5.0
3	0	NA	6*	NA	Yes	Every winter	Mustard	3x	11,783	1300	33	49	5.4
4	0	NA	6*	NA	Yes	Every winter	Mustard	3x	12,079	1400	46	50	5.8
1	0		7*	5-Compost + Rye annually	Yes	Every winter	Rye	1x	13,067	1400	53	54	5.8
2	0		7*	5-Compost + Rye annually	Yes	Every winter	Rye	1x	10,367	1100	26	43	4.6
3	0		7*	5-Compost + Rye annually	Yes	Every winter	Rye	1x	12,033	1300	33	50	5.4
4	0		7*	5-Compost + Rye annually	Yes	Every winter	Rye	1x	12,167	1300	54	51	5.4
1	0	NA	8*	NA	Yes	Every winter	Rye	3x	12,660	1300	41	53	5.4
2	0	NA	8*	NA	Yes	Every winter	Rye	3x	12,253	1300	37	51	5.4
3	0	NA	8*	NA	Yes	Every winter	Rye	3x	10,233	1100	30	43	4.6
4	0	NA	8*	NA	Yes	Every winter	Rye	3x	9533	1000	36	40	4.2
1	1		1*	1-No Compost + Legume-rye 4th Year	No	Every 4th winter	Leg-rye	3x	4975	700	20	21	2.9
2	1		1*	1-No Compost + Legume-rye 4th Year	No	Every 4th winter	Leg-rye	3x	2894	600	11	12	2.5
3	1		1*	1-No Compost + Legume-rye 4th Year	No	Every 4th winter	Leg-rye	3x	2132	500	13	9	2.1
4	1		1*	1-No Compost + Legume-rye 4th Year	No	Every 4th winter	Leg-rye	3x	4018	600	9	17	2.5
1	1		2*	2-Compost + Legume-rye 4th Year	Yes	Every 4th winter	Leg-rye	3x	6053	700	16	26	3.0
2	1		2*	2-Compost + Legume-rye 4th Year	Yes	Every 4th winter	Leg-rye	3x	4432	600	14	19	2.6
3	1		2*	2-Compost + Legume-rye 4th Year	Yes	Every 4th winter	Leg-rye	3x	2794	600	22	13	2.6
4	1		2*	2-Compost + Legume-rye 4th Year	Yes	Every 4th winter	Leg-rye	3x	6348	800	20	27	3.4
1	1	NA	3*	NA	Yes	Every winter	Leg-rye	1x	4611	600	32	20	2.6
2	1	NA	3*	NA	Yes	Every winter	Leg-rye	1x	2793	500	13	12	2.1
3	1	NA	3*	NA	Yes	Every winter	Leg-rye	1x	3756	700	17	16	3.0
4	1	NA	3*	NA	Yes	Every winter	Leg-rye	1x	8157	1000	41	34	4.2
1	1		4*	3-Compost + Legume-rye annually	Yes	Every winter	Leg-rye	3x	3957	600	25	18	2.6
2	1		4*	3-Compost + Legume-rye annually	Yes	Every winter	Leg-rye	3x	3570	600	20	16	2.6
3	1		4*	3-Compost + Legume-rye annually	Yes	Every winter	Leg-rye	3x	3312	600	21	14	2.6
4	1		4*	3-Compost + Legume-rye annually	Yes	Every winter	Leg-rye	3x	7557	1000	26	32	4.2
1	1		5*	4-Compost + Mustard annually	Yes	Every winter	Mustard	1x	2484	500	10	12	2.1
2	1		5*	4-Compost + Mustard annually	Yes	Every winter	Mustard	1x	4093	600	26	18	2.6
3	1		5*	4-Compost + Mustard annually	Yes	Every winter	Mustard	1x	2894	600	23	13	2.6
4	1		5*	4-Compost + Mustard annually	Yes	Every winter	Mustard	1x	5667	900	19	24	3.8
1	1	NA	6*	NA	Yes	Every winter	Mustard	3x	5057	600	42	22	2.6
2	1	NA	6*	NA	Yes	Every winter	Mustard	3x	3948	600	19	17	2.6
3	1	NA	6*	NA	Yes	Every winter	Mustard	3x	3256	600	12	14	2.6
4	1	NA	6*	NA	Yes	Every winter	Mustard	3x	4720	600	16	20	2.6
1	1		7*	5-Compost + Rye annually	Yes	Every winter	Rye	1x	4312	600	21	18	2.6
2	1		7*	5-Compost + Rye annually	Yes	Every winter	Rye	1x	4394	700	10	19	3.0
3	1		7*	5-Compost + Rye annually	Yes	Every winter	Rye	1x	4120	600	32	18	2.6
4	1		7*	5-Compost + Rye annually	Yes	Every winter	Rye	1x	6020	800	29	26	3.4
1	1	NA	8*	NA	Yes	Every winter	Rye	3x	4329	600	30	19	2.6
2	1	NA	8*	NA	Yes	Every winter	Rye	3x	2684	500	15	12	2.2
3	1	NA	8*	NA	Yes	Every winter	Rye	3x	2894	600	9	13	2.5
4	1	NA	8*	NA	Yes	Every winter	Rye	3x	5494	700	12	23	2.9
1	2		1*	1-No Compost + Legume-rye 4th Year	No	Every 4th winter	Leg-rye	3x	8267	800	15	34	3.3
2	2		1*	1-No Compost + Legume-rye 4th Year	No	Every 4th winter	Leg-rye	3x	7327	700	16	30	2.9
3	2		1*	1-No Compost + Legume-rye 4th Year	No	Every 4th winter	Leg-rye	3x	5446	600	19	23	2.5
4	2		1*	1-No Compost + Legume-rye 4th Year	No	Every 4th winter	Leg-rye	3x	6065	600	15	25	2.5
1	2		2*	2-Compost + Legume-rye 4th Year	Yes	Every 4th winter	Leg-rye	3x	9967	900	15	42	3.8
2	2		2*	2-Compost + Legume-rye 4th Year	Yes	Every 4th winter	Leg-rye	3x	6227	600	21	27	2.6
3	2		2*	2-Compost + Legume-rye 4th Year	Yes	Every 4th winter	Leg-rye	3x	8148	800	22	34	3.4
4	2		2*	2-Compost + Legume-rye 4th Year	Yes	Every 4th winter	Leg-rye	3x	10,371	1100	26	43	4.6
1	2	NA	3*	NA	Yes	Every winter	Leg-rye	1x	10,367	1000	27	44	4.2
2	2	NA	3*	NA	Yes	Every winter	Leg-rye	1x	9105	900	22	38	3.8
3	2	NA	3*	NA	Yes	Every winter	Leg-rye	1x	7375	700	27	31	3.0
4	2	NA	3*	NA	Yes	Every winter	Leg-rye	1x	9912	900	32	42	3.8
1	2		4*	3-Compost + Legume-rye annually	Yes	Every winter	Leg-rye	3x	9439	900	33	40	3.8
2	2		4*	3-Compost + Legume-rye annually	Yes	Every winter	Leg-rye	3x	9839	900	26	41	3.8
3	2		4*	3-Compost + Legume-rye annually	Yes	Every winter	Leg-rye	3x	8130	800	28	34	3.5
4	2		4*	3-Compost + Legume-rye annually	Yes	Every winter	Leg-rye	3x	12418	1200	39	52	5.0
1	2		5*	4-Compost + Mustard annually	Yes	Every winter	Mustard	1x	6746	600	18	29	2.6
2	2		5*	4-Compost + Mustard annually	Yes	Every winter	Mustard	1x	8342	800	31	35	3.4
3	2		5*	4-Compost + Mustard annually	Yes	Every winter	Mustard	1x	10,467	1000	23	44	4.2
4	2		5*	4-Compost + Mustard annually	Yes	Every winter	Mustard	1x	12,539	1300	29	52	5.4
1	2	NA	6*	NA	Yes	Every winter	Mustard	3x	10,275	1000	26	43	4.2
2	2	NA	6*	NA	Yes	Every winter	Mustard	3x	11,167	1200	28	46	5.0
3	2	NA	6*	NA	Yes	Every winter	Mustard	3x	7891	800	21	34	3.4
4	2	NA	6*	NA	Yes	Every winter	Mustard	3x	11,280	1200	28	47	5.0
1	2		7*	5-Compost + Rye annually	Yes	Every winter	Rye	1x	8705	800	25	36	3.5
2	2		7*	5-Compost + Rye annually	Yes	Every winter	Rye	1x	7342	700	24	31	3.0
3	2		7*	5-Compost + Rye annually	Yes	Every winter	Rye	1x	9980	900	24	42	3.9
4	2		7*	5-Compost + Rye annually	Yes	Every winter	Rye	1x	8705	900	36	37	3.9
1	2	NA	8*	NA	Yes	Every winter	Rye	3x	10,675	1000	24	45	4.2
2	2	NA	8*	NA	Yes	Every winter	Rye	3x	8030	700	26	34	3.0
3	2	NA	8*	NA	Yes	Every winter	Rye	3x	9139	700	20	38	3.0
4	2	NA	8*	NA	Yes	Every winter	Rye	3x	8748	900	23	37	3.8
1	3		1*	1-No Compost + Legume-rye 4th Year	No	Every 4th winter	Leg-rye	3x	6700	700	13	28	2.9
2	3		1*	1-No Compost + Legume-rye 4th Year	No	Every 4th winter	Leg-rye	3x	6895	600	7	29	2.5
3	3		1*	1-No Compost + Legume-rye 4th Year	No	Every 4th winter	Leg-rye	3x	5267	500	6	22	2.1
4	3		1*	1-No Compost + Legume-rye 4th Year	No	Every 4th winter	Leg-rye	3x	6344	500	3	26	2.1
1	3		2*	2-Compost + Legume-rye 4th Year	Yes	Every 4th winter	Leg-rye	3x	8275	600	5	35	2.6
2	3		2*	2-Compost + Legume-rye 4th Year	Yes	Every 4th winter	Leg-rye	3x	7933	700	6	34	3.0
3	3		2*	2-Compost + Legume-rye 4th Year	Yes	Every 4th winter	Leg-rye	3x	9263	800	8	39	3.4
4	3		2*	2-Compost + Legume-rye 4th Year	Yes	Every 4th winter	Leg-rye	3x	8067	700	9	34	3.0
1	3	NA	3*	NA	Yes	Every winter	Leg-rye	1x	9075	800	17	38	3.4
2	3	NA	3*	NA	Yes	Every winter	Leg-rye	1x	7459	800	8	32	3.4
3	3	NA	3*	NA	Yes	Every winter	Leg-rye	1x	9427	800	8	40	3.4
4	3	NA	3*	NA	Yes	Every winter	Leg-rye	1x	8486	700	14	36	3.0
1	3		4*	3-Compost + Legume-rye annually	Yes	Every winter	Leg-rye	3x	9826	800	11	42	3.4
2	3		4*	3-Compost + Legume-rye annually	Yes	Every winter	Leg-rye	3x	8337	700	9	35	3.0
3	3		4*	3-Compost + Legume-rye annually	Yes	Every winter	Leg-rye	3x	8398	700	10	35	3.1
4	3		4*	3-Compost + Legume-rye annually	Yes	Every winter	Leg-rye	3x	6483	600	10	28	2.6
1	3		5*	4-Compost + Mustard annually	Yes	Every winter	Mustard	1x	7500	700	7	32	3.0
2	3		5*	4-Compost + Mustard annually	Yes	Every winter	Mustard	1x	9156	800	12	39	3.4
3	3		5*	4-Compost + Mustard annually	Yes	Every winter	Mustard	1x	7500	600	11	32	2.6
4	3		5*	4-Compost + Mustard annually	Yes	Every winter	Mustard	1x	10,105	900	17	42	3.8
1	3	NA	6*	NA	Yes	Every winter	Mustard	3x	9663	800	13	41	3.4
2	3	NA	6*	NA	Yes	Every winter	Mustard	3x	10,463	900	18	43	3.8
3	3	NA	6*	NA	Yes	Every winter	Mustard	3x	8935	800	11	38	3.4
4	3	NA	6*	NA	Yes	Every winter	Mustard	3x	8695	700	12	37	3.0
1	3		7*	5-Compost + Rye annually	Yes	Every winter	Rye	1x	7683	700	8	32	3.1
2	3		7*	5-Compost + Rye annually	Yes	Every winter	Rye	1x	8035	700	9	34	3.0
3	3		7*	5-Compost + Rye annually	Yes	Every winter	Rye	1x	9139	800	12	38	3.5
4	3		7*	5-Compost + Rye annually	Yes	Every winter	Rye	1x	9333	800	14	40	3.5
1	3	NA	8*	NA	Yes	Every winter	Rye	3x	9521	800	12	40	3.4
2	3	NA	8*	NA	Yes	Every winter	Rye	3x	9595	900	8	41	3.8
3	3	NA	8*	NA	Yes	Every winter	Rye	3x	8696	800	13	37	3.4
4	3	NA	8*	NA	Yes	Every winter	Rye	3x	8652	700	15	36	3.0
1	4		1*	1-No Compost + Legume-rye 4th Year	No	Every 4th winter	Leg-rye	3x	5171	500	12	22	2.1
2	4		1*	1-No Compost + Legume-rye 4th Year	No	Every 4th winter	Leg-rye	3x	4333	500	11	18	2.1
3	4		1*	1-No Compost + Legume-rye 4th Year	No	Every 4th winter	Leg-rye	3x	4274	500	8	18	2.1
4	4		1*	1-No Compost + Legume-rye 4th Year	No	Every 4th winter	Leg-rye	3x	5799	600	8	24	2.5
1	4		2*	2-Compost + Legume-rye 4th Year	Yes	Every 4th winter	Leg-rye	3x	7178	800	10	31	3.4
2	4		2*	2-Compost + Legume-rye 4th Year	Yes	Every 4th winter	Leg-rye	3x	5758	600	15	26	2.7
3	4		2*	2-Compost + Legume-rye 4th Year	Yes	Every 4th winter	Leg-rye	3x	5127	500	22	23	2.2
4	4		2*	2-Compost + Legume-rye 4th Year	Yes	Every 4th winter	Leg-rye	3x	6807	700	15	29	3.0
1	4	NA	3*	NA	Yes	Every winter	Leg-rye	1x	5888	600	27	26	2.6
2	4	NA	3*	NA	Yes	Every winter	Leg-rye	1x	5051	600	10	22	2.6
3	4	NA	3*	NA	Yes	Every winter	Leg-rye	1x	5327	600	11	23	2.6
4	4	NA	3*	NA	Yes	Every winter	Leg-rye	1x	8022	800	19	34	3.4
1	4		4*	3-Compost + Legume-rye annually	Yes	Every winter	Leg-rye	3x	6188	600	22	28	2.7
2	4		4*	3-Compost + Legume-rye annually	Yes	Every winter	Leg-rye	3x	6410	700	19	28	3.1
3	4		4*	3-Compost + Legume-rye annually	Yes	Every winter	Leg-rye	3x	6599	700	19	28	3.1
4	4		4*	3-Compost + Legume-rye annually	Yes	Every winter	Leg-rye	3x	7067	700	34	31	3.1
1	4		5*	4-Compost + Mustard annually	Yes	Every winter	Mustard	1x	4420	400	11	21	1.9
2	4		5*	4-Compost + Mustard annually	Yes	Every winter	Mustard	1x	7323	700	17	32	3.1
3	4		5*	4-Compost + Mustard annually	Yes	Every winter	Mustard	1x	6781	700	11	30	3.1
4	4		5*	4-Compost + Mustard annually	Yes	Every winter	Mustard	1x	7638	800	17	33	3.5
1	4	NA	6*	NA	Yes	Every winter	Mustard	3x	6629	600	20	29	2.6
2	4	NA	6*	NA	Yes	Every winter	Mustard	3x	5735	600	10	25	2.6
3	4	NA	6*	NA	Yes	Every winter	Mustard	3x	5389	600	13	24	2.6
4	4	NA	6*	NA	Yes	Every winter	Mustard	3x	7483	700	16	32	3.0
1	4		7*	5-Compost + Rye annually	Yes	Every winter	Rye	1x	4537	500	12	21	2.4
2	4		7*	5-Compost + Rye annually	Yes	Every winter	Rye	1x	5629	600	7	25	2.7
3	4		7*	5-Compost + Rye annually	Yes	Every winter	Rye	1x	5210	600	16	24	2.7
4	4		7*	5-Compost + Rye annually	Yes	Every winter	Rye	1x	8720	800	15	37	3.5
1	4	NA	8*	NA	Yes	Every winter	Rye	3x	8740	800	17	37	3.4
2	4	NA	8*	NA	Yes	Every winter	Rye	3x	7023	700	15	30	3.0
3	4	NA	8*	NA	Yes	Every winter	Rye	3x	5858	600	12	25	2.6
4	4	NA	8*	NA	Yes	Every winter	Rye	3x	7395	700	8	31	3.0
1	5		1*	1-No Compost + Legume-rye 4th Year	No	Every 4th winter	Leg-rye	3x	5756	600	10	24	2.5
2	5		1*	1-No Compost + Legume-rye 4th Year	No	Every 4th winter	Leg-rye	3x	4798	500	9	20	2.1
3	5		1*	1-No Compost + Legume-rye 4th Year	No	Every 4th winter	Leg-rye	3x	3867	400	7	16	1.7
4	5		1*	1-No Compost + Legume-rye 4th Year	No	Every 4th winter	Leg-rye	3x	7110	700	27	30	2.9
1	5		2*	2-Compost + Legume-rye 4th Year	Yes	Every 4th winter	Leg-rye	3x	6136	600	9	27	2.6
2	5		2*	2-Compost + Legume-rye 4th Year	Yes	Every 4th winter	Leg-rye	3x	6496	600	12	29	2.7
3	5		2*	2-Compost + Legume-rye 4th Year	Yes	Every 4th winter	Leg-rye	3x	7139	700	13	31	3.0
4	5		2*	2-Compost + Legume-rye 4th Year	Yes	Every 4th winter	Leg-rye	3x	7158	700	15	31	3.0
1	5	NA	3*	NA	Yes	Every winter	Leg-rye	1x	7809	700	21	33	3.0
2	5	NA	3*	NA	Yes	Every winter	Leg-rye	1x	8936	900	19	37	3.8
3	5	NA	3*	NA	Yes	Every winter	Leg-rye	1x	6725	600	17	29	2.6
4	5	NA	3*	NA	Yes	Every winter	Leg-rye	1x	6456	600	29	28	2.6
1	5		4*	3-Compost + Legume-rye annually	Yes	Every winter	Leg-rye	3x	8686	800	23	38	3.5
2	5		4*	3-Compost + Legume-rye annually	Yes	Every winter	Leg-rye	3x	7383	700	26	32	3.1
3	5		4*	3-Compost + Legume-rye annually	Yes	Every winter	Leg-rye	3x	6394	600	20	28	2.8
4	5		4*	3-Compost + Legume-rye annually	Yes	Every winter	Leg-rye	3x	8839	800	26	38	3.4
1	5		5*	4-Compost + Mustard annually	Yes	Every winter	Mustard	1x	6577	600	13	29	2.6
2	5		5*	4-Compost + Mustard annually	Yes	Every winter	Mustard	1x	5958	600	21	27	2.7
3	5		5*	4-Compost + Mustard annually	Yes	Every winter	Mustard	1x	5835	500	18	27	2.3
4	5		5*	4-Compost + Mustard annually	Yes	Every winter	Mustard	1x	9744	900	17	41	3.9
1	5	NA	6*	NA	Yes	Every winter	Mustard	3x	9494	900	23	40	3.8
2	5	NA	6*	NA	Yes	Every winter	Mustard	3x	7856	700	20	33	3.0
3	5	NA	6*	NA	Yes	Every winter	Mustard	3x	6058	600	14	26	2.6
4	5	NA	6*	NA	Yes	Every winter	Mustard	3x	4567	500	12	20	2.2
1	5		7*	5-Compost + Rye annually	Yes	Every winter	Rye	1x	7528	700	20	32	3.2
2	5		7*	5-Compost + Rye annually	Yes	Every winter	Rye	1x	9609	900	15	40	3.8
3	5		7*	5-Compost + Rye annually	Yes	Every winter	Rye	1x	7289	700	18	31	3.1
4	5		7*	5-Compost + Rye annually	Yes	Every winter	Rye	1x	8553	800	20	37	3.5
1	5	NA	8*	NA	Yes	Every winter	Rye	3x	8232	800	22	35	3.4
2	5	NA	8*	NA	Yes	Every winter	Rye	3x	8372	800	15	36	3.4
3	5	NA	8*	NA	Yes	Every winter	Rye	3x	5880	500	10	25	2.2
4	5	NA	8*	NA	Yes	Every winter	Rye	3x	7194	700	13	30	3.0
1	6		1*	1-No Compost + Legume-rye 4th Year	No	Every 4th winter	Leg-rye	3x	6167	550	19	26	2.3
2	6		1*	1-No Compost + Legume-rye 4th Year	No	Every 4th winter	Leg-rye	3x	6983	650	21	29	2.7
3	6		1*	1-No Compost + Legume-rye 4th Year	No	Every 4th winter	Leg-rye	3x	5508	510	16	23	2.1
4	6		1*	1-No Compost + Legume-rye 4th Year	No	Every 4th winter	Leg-rye	3x	6029	550	15	25	2.3
1	6		2*	2-Compost + Legume-rye 4th Year	Yes	Every 4th winter	Leg-rye	3x	7633	670	25	33	2.9
2	6		2*	2-Compost + Legume-rye 4th Year	Yes	Every 4th winter	Leg-rye	3x	11,747	1040	31	49	4.4
3	6		2*	2-Compost + Legume-rye 4th Year	Yes	Every 4th winter	Leg-rye	3x	9579	890	29	40	3.8
4	6		2*	2-Compost + Legume-rye 4th Year	Yes	Every 4th winter	Leg-rye	3x	9093	820	22	39	3.5
1	6	NA	3*	NA	Yes	Every winter	Leg-rye	1x	9387	820	37	40	3.5
2	6	NA	3*	NA	Yes	Every winter	Leg-rye	1x	9874	880	64	41	3.7
3	6	NA	3*	NA	Yes	Every winter	Leg-rye	1x	7800	710	37	33	3.0
4	6	NA	3*	NA	Yes	Every winter	Leg-rye	1x	9006	820	33	38	3.5
1	6		4*	3-Compost + Legume-rye annually	Yes	Every winter	Leg-rye	3x	11,102	1010	38	47	4.3
2	6		4*	3-Compost + Legume-rye annually	Yes	Every winter	Leg-rye	3x	10,614	980	44	44	4.2
3	6		4*	3-Compost + Legume-rye annually	Yes	Every winter	Leg-rye	3x	9018	810	57	38	3.6
4	6		4*	3-Compost + Legume-rye annually	Yes	Every winter	Leg-rye	3x	7619	700	37	33	3.1
1	6		5*	4-Compost + Mustard annually	Yes	Every winter	Mustard	1x	7133	630	40	32	2.8
2	6		5*	4-Compost + Mustard annually	Yes	Every winter	Mustard	1x	11,433	1030	36	48	4.3
3	6		5*	4-Compost + Mustard annually	Yes	Every winter	Mustard	1x	7900	740	44	35	3.2
4	6		5*	4-Compost + Mustard annually	Yes	Every winter	Mustard	1x	9444	880	37	40	3.8
1	6	NA	6*	NA	Yes	Every winter	Mustard	3x	9383	830	33	40	3.6
2	6	NA	6*	NA	Yes	Every winter	Mustard	3x	9174	820	55	38	3.5
3	6	NA	6*	NA	Yes	Every winter	Mustard	3x	8344	790	38	35	3.4
4	6	NA	6*	NA	Yes	Every winter	Mustard	3x	8129	770	54	35	3.3
1	6		7*	5-Compost + Rye annually	Yes	Every winter	Rye	1x	8160	720	41	34	3.2
2	6		7*	5-Compost + Rye annually	Yes	Every winter	Rye	1x	11,218	1010	32	46	4.2
3	6		7*	5-Compost + Rye annually	Yes	Every winter	Rye	1x	7914	730	35	34	3.2
4	6		7*	5-Compost + Rye annually	Yes	Every winter	Rye	1x	7900	740	40	34	3.3
1	6	NA	8*	NA	Yes	Every winter	Rye	3x	9960	880	44	42	3.8
2	6	NA	8*	NA	Yes	Every winter	Rye	3x	9667	880	35	41	3.8
3	6	NA	8*	NA	Yes	Every winter	Rye	3x	8744	810	34	37	3.4
4	6	NA	8*	NA	Yes	Every winter	Rye	3x	8918	820	32	37	3.5
1	7		1*	1-No Compost + Legume-rye 4th Year	No	Every 4th winter	Leg-rye	3x	3414	320	5	15	1.4
2	7		1*	1-No Compost + Legume-rye 4th Year	No	Every 4th winter	Leg-rye	3x	4732	460	5	20	2.0
3	7		1*	1-No Compost + Legume-rye 4th Year	No	Every 4th winter	Leg-rye	3x	5125	500	5	22	2.1
4	7		1*	1-No Compost + Legume-rye 4th Year	No	Every 4th winter	Leg-rye	3x	6484	610	9	27	2.6
1	7		2*	2-Compost + Legume-rye 4th Year	Yes	Every 4th winter	Leg-rye	3x	4439	420	5	21	2.0
2	7		2*	2-Compost + Legume-rye 4th Year	Yes	Every 4th winter	Leg-rye	3x	7944	720	8	35	3.2
3	7		2*	2-Compost + Legume-rye 4th Year	Yes	Every 4th winter	Leg-rye	3x	7740	710	9	33	3.1
4	7		2*	2-Compost + Legume-rye 4th Year	Yes	Every 4th winter	Leg-rye	3x	8667	790	7	37	3.5
1	7	NA	3*	NA	Yes	Every winter	Leg-rye	1x	8475	750	20	37	3.4
2	7	NA	3*	NA	Yes	Every winter	Leg-rye	1x	9133	860	18	39	3.7
3	7	NA	3*	NA	Yes	Every winter	Leg-rye	1x	7123	650	14	31	2.9
4	7	NA	3*	NA	Yes	Every winter	Leg-rye	1x	8767	840	27	38	3.7
1	7		4*	3-Compost + Legume-rye annually	Yes	Every winter	Leg-rye	3x	7801	740	21	35	3.3
2	7		4*	3-Compost + Legume-rye annually	Yes	Every winter	Leg-rye	3x	7710	740	23	33	3.3
3	7		4*	3-Compost + Legume-rye annually	Yes	Every winter	Leg-rye	3x	12,294	1170	22	50	5.0
4	7		4*	3-Compost + Legume-rye annually	Yes	Every winter	Leg-rye	3x	9367	870	20	40	3.7
1	7		5*	4-Compost + Mustard annually	Yes	Every winter	Mustard	1x	6497	630	21	30	2.8
2	7		5*	4-Compost + Mustard annually	Yes	Every winter	Mustard	1x	14,927	1400	23	61	5.7
3	7		5*	4-Compost + Mustard annually	Yes	Every winter	Mustard	1x	7410	690	18	33	3.1
4	7		5*	4-Compost + Mustard annually	Yes	Every winter	Mustard	1x	7567	700	20	33	3.1
1	7	NA	6*	NA	Yes	Every winter	Mustard	3x	10,775	980	17	46	4.2
2	7	NA	6*	NA	Yes	Every winter	Mustard	3x	8755	810	16	37	3.5
3	7	NA	6*	NA	Yes	Every winter	Mustard	3x	8633	820	16	37	3.6
4	7	NA	6*	NA	Yes	Every winter	Mustard	3x	9333	870	25	40	3.8
1	7		7*	5-Compost + Rye annually	Yes	Every winter	Rye	1x	8630	800	17	36	3.6
2	7		7*	5-Compost + Rye annually	Yes	Every winter	Rye	1x	11,406	1060	16	47	4.4
3	7		7*	5-Compost + Rye annually	Yes	Every winter	Rye	1x	10171	970	12	42	4.2
4	7		7*	5-Compost + Rye annually	Yes	Every winter	Rye	1x	8600	850	14	37	3.7
1	7	NA	8*	NA	Yes	Every winter	Rye	3x	12,400	1160	21	52	4.9
2	7	NA	8*	NA	Yes	Every winter	Rye	3x	11,006	1020	19	46	4.4
3	7	NA	8*	NA	Yes	Every winter	Rye	3x	7839	720	15	34	3.1
4	7	NA	8*	NA	Yes	Every winter	Rye	3x	8075	720	9	34	3.1
1	8		1*	1-No Compost + Legume-rye 4th Year	No	Every 4th winter	Leg-rye	3x	5186	510	27	22	2.2
2	8		1*	1-No Compost + Legume-rye 4th Year	No	Every 4th winter	Leg-rye	3x	4006	440	29	17	1.9
3	8		1*	1-No Compost + Legume-rye 4th Year	No	Every 4th winter	Leg-rye	3x	4279	440	21	18	1.9
4	8		1*	1-No Compost + Legume-rye 4th Year	No	Every 4th winter	Leg-rye	3x	5833	590	30	24	2.5
1	8		2*	2-Compost + Legume-rye 4th Year	Yes	Every 4th winter	Leg-rye	3x	5433	530	30	24	2.4
2	8		2*	2-Compost + Legume-rye 4th Year	Yes	Every 4th winter	Leg-rye	3x	8475	850	36	37	3.7
3	8		2*	2-Compost + Legume-rye 4th Year	Yes	Every 4th winter	Leg-rye	3x	5733	540	26	26	2.5
4	8		2*	2-Compost + Legume-rye 4th Year	Yes	Every 4th winter	Leg-rye	3x	7623	760	33	33	3.3
1	8	NA	3*	NA	Yes	Every winter	Leg-rye	1x	8110	780	38	36	3.5
2	8	NA	3*	NA	Yes	Every winter	Leg-rye	1x	6867	670	35	30	3.0
3	8	NA	3*	NA	Yes	Every winter	Leg-rye	1x	6971	690	35	31	3.0
4	8	NA	3*	NA	Yes	Every winter	Leg-rye	1x	7359	720	32	32	3.2
1	8		4*	3-Compost + Legume-rye annually	Yes	Every winter	Leg-rye	3x	9210	880	50	40	3.9
2	8		4*	3-Compost + Legume-rye annually	Yes	Every winter	Leg-rye	3x	6395	640	39	28	2.9
3	8		4*	3-Compost + Legume-rye annually	Yes	Every winter	Leg-rye	3x	7090	710	48	31	3.2
4	8		4*	3-Compost + Legume-rye annually	Yes	Every winter	Leg-rye	3x	7359	730	42	32	3.2
1	8		5*	4-Compost + Mustard annually	Yes	Every winter	Mustard	1x	7520	730	37	34	3.2
2	8		5*	4-Compost + Mustard annually	Yes	Every winter	Mustard	1x	8871	830	37	38	3.6
3	8		5*	4-Compost + Mustard annually	Yes	Every winter	Mustard	1x	6001	600	30	28	2.7
4	8		5*	4-Compost + Mustard annually	Yes	Every winter	Mustard	1x	7267	700	47	32	3.1
1	8	NA	6*	NA	Yes	Every winter	Mustard	3x	8627	780	33	38	3.5
2	8	NA	6*	NA	Yes	Every winter	Mustard	3x	7603	760	35	33	3.3
3	8	NA	6*	NA	Yes	Every winter	Mustard	3x	6767	690	40	30	3.1
4	8	NA	6*	NA	Yes	Every winter	Mustard	3x	6991	680	35	31	3.1
1	8		7*	5-Compost + Rye annually	Yes	Every winter	Rye	1x	6448	610	36	28	2.9
2	8		7*	5-Compost + Rye annually	Yes	Every winter	Rye	1x	6903	680	38	30	3.0
3	8		7*	5-Compost + Rye annually	Yes	Every winter	Rye	1x	8400	800	36	36	3.5
4	8		7*	5-Compost + Rye annually	Yes	Every winter	Rye	1x	10,200	920	40	43	4.0
1	8	NA	8*	NA	Yes	Every winter	Rye	3x	7855	740	38	35	3.3
2	8	NA	8*	NA	Yes	Every winter	Rye	3x	6348	610	34	29	2.8
3	8	NA	8*	NA	Yes	Every winter	Rye	3x	7401	720	33	32	3.1
4	8	NA	8*	NA	Yes	Every winter	Rye	3x	7567	750	27	32	3.2

1 The data provided in this table is from the Salinas Organic Cropping Systems (SOCS) study in Salinas, California. This includes soil total organic carbon, total nitrogen and nitrate-N data for all 8 systems in the SOCS study at the beginning of the study (year 0) and for subsequent 8 years. However, the analysis for only 5 systems with optimal seeding rates for weed suppression were included in the related article in PLoS ONE [1]. The experimental design was a randomized complete block with 4 blocks (i.e., replicates). These data are provided to give readers an opportunity use the data for future meta-analyses, or analysis of confidence intervals, effect sizes, etc. in the Explanatory Software for Confidence Intervals (ESCI) produced by Geoff Cumming. ESCI is freely available at https://thenewstatistics.com/itns/esci/

2 To account for changes in soil bulk density over time, organic carbon and nitrogen stocks were calculated using the Maximum Equivalent Soil Mass Method [7].

3 The symbols, shapes, and colors used in the PLoS ONE article. Note that in the PLoS ONE article the data for only 5 systems were included, but in this Data in Brief article, the data for all 8 systems is included. NA = not applicable because the system was not included in the PLoS ONE article.

4 In this *Data in Brief* article, these numbers (1 to 8) are used for the 8 systems.

5 In the PLoS ONE article only 5 systems with seeding rates that provided optimal weed suppression were included. NA= not applicable because these 3 systems were not included in the PLoS ONE article.

6 The application rate for compost, which was applied prior to each vegetable crop, was 7.6 Mg ha^−1^ on an oven dry weight basis. The compost was made from urban yard waste.

7 Winter cover cropping period was from October or November to February or March.

8 See Table 1 for details on the cover crop types and seeding rates.

**Table 3:** Raw data of cumulative cover crop and vegetable carbon inputs, legume nitrogen fixation, cover crop and vegetable crop N uptake and export during 8 years at the Salinas Organic Cropping Systems experiment in Salinas, California. This includes data from all eight systems in the experiment. The related article in PLoS ONE [1] only included data from five of the eight systems with optimal seeding rates for weed suppression. A Microsoft Excel version of the table is available in the supplementary material (Supplementary Table 2).

Overview of the data^1^								Cumulative Plant Carbon and Nitrogen Inputs							Cumulative Nitrogen Uptake and Export					
Block (i.e. replicate)	Symbol color & shape in PloS One article figures^2^	System ID in *Data in Brief* article^3^	System ID & description used in associated article in *PLoS ONE*^4^	Compost added^5^	Winer cover cropping frequency^6^	Cover crop type^7^	Cover crop seeding rate^7^	Cover Crop Shoot C	Cover Crop Root C	Cover Crop Root Exudate C	Vegetable Shoot Residue C	Vegetable Root C	Vegetable Root Exudate C	Legume N Fixation	Cover Crop N Uptake	Vegetable Residue N	Lettuce N Uptake	Broccoli N Uptake	N Export in Lettuce Harvest	N Export in Broccoli Harvest
								Mg ha^−1^						kg ha^−1^						
1		1*	1-No Compost + Legume-rye 4th Year	No	Every 4th winter	Leg-rye	3x	5.45	1.06	0.691	17.7	5.23	3.40	135	169	1298	534	1182	116	303
2		1*	1-No Compost + Legume-rye 4th Year	No	Every 4th winter	Leg-rye	3x	6.02	1.16	0.756	17.1	5.07	3.30	132	167	1295	511	1222	111	327
3		1*	1-No Compost + Legume-rye 4th Year	No	Every 4th winter	Leg-rye	3x	6.78	1.31	0.852	16.5	4.89	3.18	149	199	1306	493	1248	107	328
4		1*	1-No Compost + Legume-rye 4th Year	No	Every 4th winter	Leg-rye	3x	6.92	1.34	0.869	16.3	4.83	3.14	150	190	1269	470	1223	102	322
1		2*	2-Compost + Legume-rye 4th Year	Yes	Every 4th winter	Leg-rye	3x	6.71	1.29	0.838	16.9	5.04	3.27	134	220	1257	544	1130	118	298
2		2*	2-Compost + Legume-rye 4th Year	Yes	Every 4th winter	Leg-rye	3x	6.97	1.35	0.875	18.2	5.42	3.52	149	216	1491	656	1324	143	346
3		2*	2-Compost + Legume-rye 4th Year	Yes	Every 4th winter	Leg-rye	3x	6.52	1.26	0.821	18.9	5.63	3.66	147	202	1525	654	1369	142	356
4		2*	2-Compost + Legume-rye 4th Year	Yes	Every 4th winter	Leg-rye	3x	6.30	1.23	0.801	18.0	5.35	3.48	159	202	1502	648	1348	141	353
1	NA	3*	NA	Yes	Every winter	Leg-rye	1x	23.5	4.46	2.90	19.4	5.75	3.74	387	933	1990	886	1719	193	423
2	NA	3*	NA	Yes	Every winter	Leg-rye	1x	25.0	4.79	3.11	19.0	5.64	3.66	482	875	1741	859	1449	187	381
3	NA	3*	NA	Yes	Every winter	Leg-rye	1x	24.9	4.68	3.04	19.9	5.89	3.83	360	967	1855	853	1599	186	412
4	NA	3*	NA	Yes	Every winter	Leg-rye	1x	23.8	4.44	2.88	20.9	6.18	4.02	281	1102	2119	879	1912	191	481
1		4*	3-Compost + Legume-rye annually	Yes	Every winter	Leg-rye	3x	25.5	4.88	3.17	19.7	5.89	3.83	481	1145	1960	965	1621	210	416
2		4*	3-Compost + Legume-rye annually	Yes	Every winter	Leg-rye	3x	25.8	4.86	3.16	21.0	6.26	4.07	379	1054	2077	900	1883	196	510
3		4*	3-Compost + Legume-rye annually	Yes	Every winter	Leg-rye	3x	27.5	5.29	3.44	20.9	6.25	4.06	567	1033	2009	952	1705	207	441
4		4*	3-Compost + Legume-rye annually	Yes	Every winter	Leg-rye	3x	26.9	5.06	3.29	19.9	5.93	3.85	371	1074	1992	889	1750	193	454
1		5*	4-Compost + Mustard annually	Yes	Every winter	Mustard	1x	16.5	2.62	1.71	18.1	5.39	3.50	0	805	1551	724	1339	157	354
2		5*	4-Compost + Mustard annually	Yes	Every winter	Mustard	1x	19.1	3.03	1.97	18.8	5.61	3.65	0	1027	1833	808	1631	176	430
3		5*	4-Compost + Mustard annually	Yes	Every winter	Mustard	1x	18.5	2.94	1.91	20.6	6.13	3.98	0	1053	1846	823	1614	179	412
4		5*	4-Compost + Mustard annually	Yes	Every winter	Mustard	1x	20.2	3.21	2.09	20.4	6.05	3.93	0	1243	1969	842	1786	183	476
1	NA	6*	NA	Yes	Every winter	Mustard	3x	22.1	3.94	2.56	18.9	5.60	3.64	0	1332	1871	872	1616	190	427
2	NA	6*	NA	Yes	Every winter	Mustard	3x	21.7	3.88	2.52	21.0	6.18	4.02	0	1035	1871	831	1646	181	425
3	NA	6*	NA	Yes	Every winter	Mustard	3x	18.0	3.21	2.09	19.3	5.71	3.71	0	870	1712	826	1444	180	379
4	NA	6*	NA	Yes	Every winter	Mustard	3x	19.3	3.44	2.24	20.1	5.94	3.86	0	1036	1851	876	1588	190	421
1		7*	5-Compost + Rye annually	Yes	Every winter	Rye	1x	24.4	4.37	2.84	18.7	5.59	3.63	0	1023	1616	803	1328	175	340
2		7*	5-Compost + Rye annually	Yes	Every winter	Rye	1x	23.1	4.13	2.68	17.9	5.36	3.48	0	831	1538	726	1321	158	352
3		7*	5-Compost + Rye annually	Yes	Every winter	Rye	1x	24.6	4.39	2.85	20.3	6.03	3.92	0	1016	1765	787	1553	171	403
4		7*	5-Compost + Rye annually	Yes	Every winter	Rye	1x	28.6	5.11	3.32	20.6	6.13	3.98	0	1272	1909	861	1665	187	430
1	NA	8*	NA	Yes	Every winter	Rye	3x	25.7	4.59	2.98	19.6	5.80	3.77	0	1104	1814	835	1565	182	404
2	NA	8*	NA	Yes	Every winter	Rye	3x	23.6	4.21	2.74	19.6	5.79	3.77	0	994	1698	817	1436	178	377
3	NA	8*	NA	Yes	Every winter	Rye	3x	24.4	4.35	2.83	18.6	5.51	3.58	0	891	1633	724	1441	157	375
4	NA	8*	NA	Yes	Every winter	Rye	3x	27.8	4.96	3.22	19.5	5.76	3.74	0	1084	1641	765	1411	166	368

1 The data provided in this table is from the Salinas Organic Cropping Systems (SOCS) study in Salinas, California. This includes cumulative cover crop and vegetable carbon inputs, legume nitrogen fixation, cover crop and vegetable crop N uptake and export for all 8 systems in the SOCS study over 8 years. However, the analysis for only 5 systems with optimal seeding rates for weed suppression were included in the related article in PLoS ONE [1]. The experimental design was a randomized complete block with 4 blocks (i.e., replicates). These data are provided to give readers an opportunity use the data for future meta-analyses, or analysis of confidence intervals, effect sizes, etc. in the Explanatory Software for Confidence Intervals (ESCI) produced by Geoff Cumming. ESCI is freely available at https://thenewstatistics.com/itns/esci/

2 The symbols, shapes, and colors used in the PLoS ONE article. Note that in the PLoS ONE article the data for only 5 systems were included, but in this Data in Brief article, the data for all 8 systems is included. NA= not applicable because the system was not included in the PLoS ONE article.

3 In this *Data in Brief* article, these numbers (1–8) are used for the 8 systems.

4 In the PLoS ONE article only 5 systems with seeding rates that provided optimal weed suppression were included. NA= not applicable because these 3 systems were not included in the PLoS ONE article.

5 The application rate for compost, which was applied prior to each vegetable crop, was 7.6 Mg ha^−1^ on an oven dry weight basis. The compost was made from urban yard waste.

6 Winter cover cropping period was from October or November to February or March.

7 See Table 1 for details on the cover crop types and seeding rates.

**Table 4:** Raw data of soil permanganate oxidizable carbon (POX-C) concentrations and stocks at the 0 to 6.7 cm depth in years 0 and 6, and the 0 to 30 cm depth in year 8 from the Salinas Organic Cropping Systems experiment in Salinas, California This data from five of the eight systems with optimal seeding rates for weed suppression was included the related paper in PLoS ONE [1]. A Microsoft Excel version of the table is available in the supplementary material (Supplementary Table 3).

Overview of the data^1^										Labile Carbon^2^	
Block (i.e. replicate)	Year	Symbol color & shape in PLoS ONE article figures^3^	System ID in *Data in Brief* article^4^	System ID & description used in associated article in *PLoS ONE*^5^	Compost added^6^	Winer cover cropping frequency^7^	Cover crop type^8^	Cover crop seeding rate^8^	Sample Depth	POX-C Concentration	POX-C Stock
									cm	mg kg^−1^	Mg ha^−1^
1	0		1*	1-No Compost + Legume-rye 4th Year	No	Every 4th winter	Leg-rye	3x	0 to 6.5	316	0.293
2	0		1*	1-No Compost + Legume-rye 4th Year	No	Every 4th winter	Leg-rye	3x	0 to 6.5	327	0.303
3	0		1*	1-No Compost + Legume-rye 4th Year	No	Every 4th winter	Leg-rye	3x	0 to 6.5	321	0.298
4	0		1*	1-No Compost + Legume-rye 4th Year	No	Every 4th winter	Leg-rye	3x	0 to 6.5	324	0.301
1	0		2*	2-Compost + Legume-rye 4th Year	Yes	Every 4th winter	Leg-rye	3x	0 to 6.5	316	0.293
2	0		2*	2-Compost + Legume-rye 4th Year	Yes	Every 4th winter	Leg-rye	3x	0 to 6.5	321	0.298
3	0		2*	2-Compost + Legume-rye 4th Year	Yes	Every 4th winter	Leg-rye	3x	0 to 6.5	341	0.316
4	0		2*	2-Compost + Legume-rye 4th Year	Yes	Every 4th winter	Leg-rye	3x	0 to 6.5	322	0.299
1	0	NA	3*	NA	Yes	Every winter	Leg-rye	1x	0 to 6.5	NA	NA
2	0	NA	3*	NA	Yes	Every winter	Leg-rye	1x	0 to 6.5	NA	NA
3	0	NA	3*	NA	Yes	Every winter	Leg-rye	1x	0 to 6.5	NA	NA
4	0	NA	3*	NA	Yes	Every winter	Leg-rye	1x	0 to 6.5	NA	NA
1	0		4*	3-Compost + Legume-rye annually	Yes	Every winter	Leg-rye	3x	0 to 6.5	319	0.296
2	0		4*	3-Compost + Legume-rye annually	Yes	Every winter	Leg-rye	3x	0 to 6.5	364	0.338
3	0		4*	3-Compost + Legume-rye annually	Yes	Every winter	Leg-rye	3x	0 to 6.5	363	0.337
4	0		4*	3-Compost + Legume-rye annually	Yes	Every winter	Leg-rye	3x	0 to 6.5	362	0.336
1	0		5*	4-Compost + Mustard annually	Yes	Every winter	Mustard	1x	0 to 6.5	252	0.234
2	0		5*	4-Compost + Mustard annually	Yes	Every winter	Mustard	1x	0 to 6.5	318	0.295
3	0		5*	4-Compost + Mustard annually	Yes	Every winter	Mustard	1x	0 to 6.5	317	0.295
4	0		5*	4-Compost + Mustard annually	Yes	Every winter	Mustard	1x	0 to 6.5	354	0.329
1	0	NA	6*	NA	Yes	Every winter	Mustard	3x	0 to 6.5	NA	NA
2	0	NA	6*	NA	Yes	Every winter	Mustard	3x	0 to 6.5	NA	NA
3	0	NA	6*	NA	Yes	Every winter	Mustard	3x	0 to 6.5	NA	NA
4	0	NA	6*	NA	Yes	Every winter	Mustard	3x	0 to 6.5	NA	NA
1	0		7*	5-Compost + Rye annually	Yes	Every winter	Rye	1x	0 to 6.5	403	0.37
2	0		7*	5-Compost + Rye annually	Yes	Every winter	Rye	1x	0 to 6.5	365	0.34
3	0		7*	5-Compost + Rye annually	Yes	Every winter	Rye	1x	0 to 6.5	413	0.38
4	0		7*	5-Compost + Rye annually	Yes	Every winter	Rye	1x	0 to 6.5	390	0.36
1	0	NA	8*	NA	Yes	Every winter	Rye	3x	0 to 6.5	NA	NA
2	0	NA	8*	NA	Yes	Every winter	Rye	3x	0 to 6.5	NA	NA
3	0	NA	8*	NA	Yes	Every winter	Rye	3x	0 to 6.5	NA	NA
4	0	NA	8*	NA	Yes	Every winter	Rye	3x	0 to 6.5	NA	NA
1	6		1*	1-No Compost + Legume-rye 4th Year	No	Every 4th winter	Leg-rye	3x	0 to 6.5	349	0.324
2	6		1*	1-No Compost + Legume-rye 4th Year	No	Every 4th winter	Leg-rye	3x	0 to 6.5	369	0.343
3	6		1*	1-No Compost + Legume-rye 4th Year	No	Every 4th winter	Leg-rye	3x	0 to 6.5	342	0.318
4	6		1*	1-No Compost + Legume-rye 4th Year	No	Every 4th winter	Leg-rye	3x	0 to 6.5	360	0.334
1	6		2*	2-Compost + Legume-rye 4th Year	Yes	Every 4th winter	Leg-rye	3x	0 to 6.5	366	0.339
2	6		2*	2-Compost + Legume-rye 4th Year	Yes	Every 4th winter	Leg-rye	3x	0 to 6.5	498	0.462
3	6		2*	2-Compost + Legume-rye 4th Year	Yes	Every 4th winter	Leg-rye	3x	0 to 6.5	417	0.388
4	6		2*	2-Compost + Legume-rye 4th Year	Yes	Every 4th winter	Leg-rye	3x	0 to 6.5	479	0.445
1	6	NA	3*	NA	Yes	Every winter	Leg-rye	1x	0 to 6.5	NA	NA
2	6	NA	3*	NA	Yes	Every winter	Leg-rye	1x	0 to 6.5	NA	NA
3	6	NA	3*	NA	Yes	Every winter	Leg-rye	1x	0 to 6.5	NA	NA
4	6	NA	3*	NA	Yes	Every winter	Leg-rye	1x	0 to 6.5	NA	NA
1	6		4*	3-Compost + Legume-rye annually	Yes	Every winter	Leg-rye	3x	0 to 6.5	551	0.512
2	6		4*	3-Compost + Legume-rye annually	Yes	Every winter	Leg-rye	3x	0 to 6.5	552	0.513
3	6		4*	3-Compost + Legume-rye annually	Yes	Every winter	Leg-rye	3x	0 to 6.5	609	0.566
4	6		4*	3-Compost + Legume-rye annually	Yes	Every winter	Leg-rye	3x	0 to 6.5	546	0.507
1	6		5*	4-Compost + Mustard annually	Yes	Every winter	Mustard	1x	0 to 6.5	523	0.486
2	6		5*	4-Compost + Mustard annually	Yes	Every winter	Mustard	1x	0 to 6.5	574	0.533
3	6		5*	4-Compost + Mustard annually	Yes	Every winter	Mustard	1x	0 to 6.5	594	0.551
4	6		5*	4-Compost + Mustard annually	Yes	Every winter	Mustard	1x	0 to 6.5	611	0.568
1	6	NA	6*	NA	Yes	Every winter	Mustard	3x	0 to 6.5	NA	NA
2	6	NA	6*	NA	Yes	Every winter	Mustard	3x	0 to 6.5	NA	NA
3	6	NA	6*	NA	Yes	Every winter	Mustard	3x	0 to 6.5	NA	NA
4	6	NA	6*	NA	Yes	Every winter	Mustard	3x	0 to 6.5	NA	NA
1	6		7*	5-Compost + Rye annually	Yes	Every winter	Rye	1x	0 to 6.5	601	0.558
2	6		7*	5-Compost + Rye annually	Yes	Every winter	Rye	1x	0 to 6.5	551	0.512
3	6		7*	5-Compost + Rye annually	Yes	Every winter	Rye	1x	0 to 6.5	497	0.461
4	6		7*	5-Compost + Rye annually	Yes	Every winter	Rye	1x	0 to 6.5	589	0.546
1	6	NA	8*	NA	Yes	Every winter	Rye	3x	0 to 6.5	NA	NA
2	6	NA	8*	NA	Yes	Every winter	Rye	3x	0 to 6.5	NA	NA
3	6	NA	8*	NA	Yes	Every winter	Rye	3x	0 to 6.5	NA	NA
4	6	NA	8*	NA	Yes	Every winter	Rye	3x	0 to 6.5	NA	NA
1	8		1*	1-No Compost + Legume-rye 4th Year	No	Every 4th winter	Leg-rye	3x	0 to 30	393	1.63
2	8		1*	1-No Compost + Legume-rye 4th Year	No	Every 4th winter	Leg-rye	3x	0 to 30	426	1.77
3	8		1*	1-No Compost + Legume-rye 4th Year	No	Every 4th winter	Leg-rye	3x	0 to 30	365	1.52
4	8		1*	1-No Compost + Legume-rye 4th Year	No	Every 4th winter	Leg-rye	3x	0 to 30	351	1.46
1	8		2*	2-Compost + Legume-rye 4th Year	Yes	Every 4th winter	Leg-rye	3x	0 to 30	356	1.48
2	8		2*	2-Compost + Legume-rye 4th Year	Yes	Every 4th winter	Leg-rye	3x	0 to 30	516	2.15
3	8		2*	2-Compost + Legume-rye 4th Year	Yes	Every 4th winter	Leg-rye	3x	0 to 30	486	2.02
4	8		2*	2-Compost + Legume-rye 4th Year	Yes	Every 4th winter	Leg-rye	3x	0 to 30	443	1.84
1	8	NA	3*	NA	Yes	Every winter	Leg-rye	1x	0 to 30	NA	NA
2	8	NA	3*	NA	Yes	Every winter	Leg-rye	1x	0 to 30	NA	NA
3	8	NA	3*	NA	Yes	Every winter	Leg-rye	1x	0 to 30	NA	NA
4	8	NA	3*	NA	Yes	Every winter	Leg-rye	1x	0 to 30	NA	NA
1	8		4*	3-Compost + Legume-rye annually	Yes	Every winter	Leg-rye	3x	0 to 30	578	2.40
2	8		4*	3-Compost + Legume-rye annually	Yes	Every winter	Leg-rye	3x	0 to 30	565	2.35
3	8		4*	3-Compost + Legume-rye annually	Yes	Every winter	Leg-rye	3x	0 to 30	592	2.46
4	8		4*	3-Compost + Legume-rye annually	Yes	Every winter	Leg-rye	3x	0 to 30	534	2.22
1	8		5*	4-Compost + Mustard annually	Yes	Every winter	Mustard	1x	0 to 30	492	2.04
2	8		5*	4-Compost + Mustard annually	Yes	Every winter	Mustard	1x	0 to 30	576	2.39
3	8		5*	4-Compost + Mustard annually	Yes	Every winter	Mustard	1x	0 to 30	573	2.38
4	8		5*	4-Compost + Mustard annually	Yes	Every winter	Mustard	1x	0 to 30	528	2.20
1	8	NA	6*	NA	Yes	Every winter	Mustard	3x	0 to 30	NA	NA
2	8	NA	6*	NA	Yes	Every winter	Mustard	3x	0 to 30	NA	NA
3	8	NA	6*	NA	Yes	Every winter	Mustard	3x	0 to 30	NA	NA
4	8	NA	6*	NA	Yes	Every winter	Mustard	3x	0 to 30	NA	NA
1	8		7*	5-Compost + Rye annually	Yes	Every winter	Rye	1x	0 to 30	578	2.40
2	8		7*	5-Compost + Rye annually	Yes	Every winter	Rye	1x	0 to 30	558	2.32
3	8		7*	5-Compost + Rye annually	Yes	Every winter	Rye	1x	0 to 30	562	2.34
4	8		7*	5-Compost + Rye annually	Yes	Every winter	Rye	1x	0 to 30	598	2.49
1	8	NA	8*	NA	Yes	Every winter	Rye	3x	0 to 30	NA	NA
2	8	NA	8*	NA	Yes	Every winter	Rye	3x	0 to 30	NA	NA
3	8	NA	8*	NA	Yes	Every winter	Rye	3x	0 to 30	NA	NA
4	8	NA	8*	NA	Yes	Every winter	Rye	3x	0 to 30	NA	NA
1	Change over 6 yrs		1*	1-No Compost + Legume-rye 4th Year	No	Every 4th winter	Leg-rye	3x	0 to 6.5	34	0.031
2	Change over 6 yrs		1*	1-No Compost + Legume-rye 4th Year	No	Every 4th winter	Leg-rye	3x	0 to 6.5	43	0.039
3	Change over 6 yrs		1*	1-No Compost + Legume-rye 4th Year	No	Every 4th winter	Leg-rye	3x	0 to 6.5	21	0.020
4	Change over 6 yrs		1*	1-No Compost + Legume-rye 4th Year	No	Every 4th winter	Leg-rye	3x	0 to 6.5	36	0.033
1	Change over 6 yrs		2*	2-Compost + Legume-rye 4th Year	Yes	Every 4th winter	Leg-rye	3x	0 to 6.5	50	0.046
2	Change over 6 yrs		2*	2-Compost + Legume-rye 4th Year	Yes	Every 4th winter	Leg-rye	3x	0 to 6.5	177	0.164
3	Change over 6 yrs		2*	2-Compost + Legume-rye 4th Year	Yes	Every 4th winter	Leg-rye	3x	0 to 6.5	77	0.071
4	Change over 6 yrs		2*	2-Compost + Legume-rye 4th Year	Yes	Every 4th winter	Leg-rye	3x	0 to 6.5	157	0.146
1	Change over 6 yrs	NA	3*	NA	Yes	Every winter	Leg-rye	1x	0 to 6.5	NA	NA
2	Change over 6 yrs	NA	3*	NA	Yes	Every winter	Leg-rye	1x	0 to 6.5	NA	NA
3	Change over 6 yrs	NA	3*	NA	Yes	Every winter	Leg-rye	1x	0 to 6.5	NA	NA
4	Change over 6 yrs	NA	3*	NA	Yes	Every winter	Leg-rye	1x	0 to 6.5	NA	NA
1	Change over 6 yrs		4*	3-Compost + Legume-rye annually	Yes	Every winter	Leg-rye	3x	0 to 6.5	233	0.216
2	Change over 6 yrs		4*	3-Compost + Legume-rye annually	Yes	Every winter	Leg-rye	3x	0 to 6.5	188	0.175
3	Change over 6 yrs		4*	3-Compost + Legume-rye annually	Yes	Every winter	Leg-rye	3x	0 to 6.5	247	0.229
4	Change over 6 yrs		4*	3-Compost + Legume-rye annually	Yes	Every winter	Leg-rye	3x	0 to 6.5	184	0.171
1	Change over 6 yrs		5*	4-Compost + Mustard annually	Yes	Every winter	Mustard	1x	0 to 6.5	271	0.251
2	Change over 6 yrs		5*	4-Compost + Mustard annually	Yes	Every winter	Mustard	1x	0 to 6.5	257	0.238
3	Change over 6 yrs		5*	4-Compost + Mustard annually	Yes	Every winter	Mustard	1x	0 to 6.5	276	0.257
4	Change over 6 yrs		5*	4-Compost + Mustard annually	Yes	Every winter	Mustard	1x	0 to 6.5	257	0.239
1	Change over 6 yrs	NA	6*	NA	Yes	Every winter	Mustard	3x	0 to 6.5	NA	NA
2	Change over 6 yrs	NA	6*	NA	Yes	Every winter	Mustard	3x	0 to 6.5	NA	NA
3	Change over 6 yrs	NA	6*	NA	Yes	Every winter	Mustard	3x	0 to 6.5	NA	NA
4	Change over 6 yrs	NA	6*	NA	Yes	Every winter	Mustard	3x	0 to 6.5	NA	NA
1	Change over 6 yrs		7*	5-Compost + Rye annually	Yes	Every winter	Rye	1x	0 to 6.5	198	0.184
2	Change over 6 yrs		7*	5-Compost + Rye annually	Yes	Every winter	Rye	1x	0 to 6.5	186	0.173
3	Change over 6 yrs		7*	5-Compost + Rye annually	Yes	Every winter	Rye	1x	0 to 6.5	83	0.077
4	Change over 6 yrs		7*	5-Compost + Rye annually	Yes	Every winter	Rye	1x	0 to 6.5	199	0.184
1	Change over 6 yrs	NA	8*	NA	Yes	Every winter	Rye	3x	0 to 6.5	NA	NA
2	Change over 6 yrs	NA	8*	NA	Yes	Every winter	Rye	3x	0 to 6.5	NA	NA
3	Change over 6 yrs	NA	8*	NA	Yes	Every winter	Rye	3x	0 to 6.5	NA	NA
4	Change over 6 yrs	NA	8*	NA	Yes	Every winter	Rye	3x	0 to 6.5	NA	NA

1 The data provided in this table is from the Salinas Organic Cropping Systems (SOCS) study in Salinas, California. This includes soil POX-C concentrations and stocks at time 0, years 6 and 8, and the change over first 6 years for the 5 systems with optimal seeding rates for weed suppression included in the related article in PLoS ONE [1]. The experimental design was a randomized complete block with 4 blocks (i.e., replicates). These data are provided to give readers an opportunity use the data for future meta-analyses, or analysis of confidence intervals, effect sizes, etc. in the Explanatory Software for Confidence Intervals (ESCI) produced by Geoff Cumming. ESCI is freely available at https://thenewstatistics.com/itns/esci/

2 To account for changes in soil bulk density over time POX-C stocks were calculated using the Maximum Equivalent Soil Mass Method [7].

3 The symbols, shapes, and colors used in the PLoS ONE article. Note that in this article the data for only 5 systems were included, but in this Data in Brief article, the data for all 8 systems is included. NA= not applicable because the system was not included in the PLoS ONE article.

4 In this *Data in Brief* article, these numbers (1–8) were uses for the 8 systems.

5 In the PLoS ONE article only 5 systems with seeding rates that provided optimal weed suppression were included. NA= not applicable because these 3 systems were not included in the PLoS ONE article.

6 The application rate for compost, which was applied prior to each vegetable crop, was 7.6 Mg ha^−1^ on an oven dry weight basis. The compost was made from urban yard waste.

7 Winter cover cropping period was from October or November to February or March.

8 See Table 1 for details on the cover crop types and seeding rates.

**Table 5:** Raw data of cumulative, estimated yields of lettuce and broccoli crop during 8 years at the Salinas Organic Cropping Systems experiment in Salinas, California; yields are on an oven-dry basis. This includes data from all eight systems in the experiment. A Microsoft Excel version of the table is available in the supplementary material (Supplementary Table 4).

Overview of the data^1^								Cumulative Estimated Yields	
Block (i.e. replicate)	Symbol color & shape in PloS One article figures^2^	System ID in *Data in Brief* article^3^	System ID & description used in associated article in *PLoS ONE*^4^	Compost added^5^	Winer cover cropping frequency^6^	Cover crop type^7^	Cover crop seeding rate^7^	Lettuce Yield	Broccoli Yield
								kg ha^−1^	
1		1*	1-No Compost + Legume-rye 4th Year	No	Every 4th winter	Leg-rye	3x	5299	8511
2		1*	1-No Compost + Legume-rye 4th Year	No	Every 4th winter	Leg-rye	3x	5380	8607
3		1*	1-No Compost + Legume-rye 4th Year	No	Every 4th winter	Leg-rye	3x	5150	7805
4		1*	1-No Compost + Legume-rye 4th Year	No	Every 4th winter	Leg-rye	3x	4890	8110
1		2*	2-Compost + Legume-rye 4th Year	Yes	Every 4th winter	Leg-rye	3x	5510	8013
2		2*	2-Compost + Legume-rye 4th Year	Yes	Every 4th winter	Leg-rye	3x	6053	8487
3		2*	2-Compost + Legume-rye 4th Year	Yes	Every 4th winter	Leg-rye	3x	6108	9021
4		2*	2-Compost + Legume-rye 4th Year	Yes	Every 4th winter	Leg-rye	3x	5981	8370
1	NA	3*	NA	Yes	Every winter	Leg-rye	1x	6583	8368
2	NA	3*	NA	Yes	Every winter	Leg-rye	1x	6765	8676
3	NA	3*	NA	Yes	Every winter	Leg-rye	1x	6816	8942
4	NA	3*	NA	Yes	Every winter	Leg-rye	1x	6872	9524
1		4*	3-Compost + Legume-rye annually	Yes	Every winter	Leg-rye	3x	7289	8426
2		4*	3-Compost + Legume-rye annually	Yes	Every winter	Leg-rye	3x	7030	10271
3		4*	3-Compost + Legume-rye annually	Yes	Every winter	Leg-rye	3x	7362	9397
4		4*	3-Compost + Legume-rye annually	Yes	Every winter	Leg-rye	3x	6831	8845
1		5*	4-Compost + Mustard annually	Yes	Every winter	Mustard	1x	6276	8324
2		5*	4-Compost + Mustard annually	Yes	Every winter	Mustard	1x	6761	8213
3		5*	4-Compost + Mustard annually	Yes	Every winter	Mustard	1x	6752	9315
4		5*	4-Compost + Mustard annually	Yes	Every winter	Mustard	1x	6563	9829
1	NA	6*	NA	Yes	Every winter	Mustard	3x	6645	8546
2	NA	6*	NA	Yes	Every winter	Mustard	3x	6717	9944
3	NA	6*	NA	Yes	Every winter	Mustard	3x	6518	8947
4	NA	6*	NA	Yes	Every winter	Mustard	3x	6842	9409
1		7*	5-Compost + Rye annually	Yes	Every winter	Rye	1x	6682	8364
2		7*	5-Compost + Rye annually	Yes	Every winter	Rye	1x	6558	7989
3		7*	5-Compost + Rye annually	Yes	Every winter	Rye	1x	6711	9463
4		7*	5-Compost + Rye annually	Yes	Every winter	Rye	1x	6859	9559
1	NA	8*	NA	Yes	Every winter	Rye	3x	6879	8698
2	NA	8*	NA	Yes	Every winter	Rye	3x	6836	8833
3	NA	8*	NA	Yes	Every winter	Rye	3x	6509	8328
4	NA	8*	NA	Yes	Every winter	Rye	3x	6520	9125

1 The data provided in this table is from the Salinas Organic Cropping Systems (SOCS) study in Salinas, California. This includes cumulative, estimated lettuce and broccoli crop yields for all 8 systems in the SOCS study. The experimental design was a randomized complete block with 4 blocks (i.e., replicates). These data are provided to give readers an opportunity use the data for future meta-analyses, or analysis of confidence intervals, effect sizes, etc. in the Explanatory Software for Confidence Intervals (ESCI) produced by Geoff Cumming. ESCI is freely available at https://thenewstatistics.com/itns/esci/

2 The symbols, shapes, and colors used in the PLoS ONE article [1]. Note that in this article the data for only 5 systems were included, but in this Data in Brief article, the data for all 8 systems is included. NA= not applicable because the system was not included in the PLoS ONE article.

3 In this *Data in Brief* article, these numbers (1 to 8) are used for the 8 systems.

4 In the PLoS ONE article only 5 systems with seeding rates that provided optimal weed suppression were included. NA= not applicable because these 3 systems were not included in the PLoS ONE article.

5 The application rate for compost, which was applied prior to each vegetable crop, was 7.6 Mg ha^−1^ on an oven dry weight basis. The compost was made from urban yard waste.

6 Winter cover cropping period was from October or November to February or March.

7 See Table 1 for details on the cover crop types and seeding rates.

## CRediT Author Statement

Kathryn E. White: Conceptualization, Data curation, Formal analysis, Investigation, Methodology, Visualization, Writing – original draft, Writing – review and editing.

Eric B. Brennan: Conceptualization, Data curation, Formal analysis, Investigation, Methodology, Project administration, Resources, Supervision, Visualization, Writing – original draft, Writing – review and editing.

Michel A. Cavigelli: Conceptualization, Methodology, Resources, Supervision, Writing – review and editing.

## Declaration of Competing Interest

The authors declare that they have no known competing financial interests or personal relationships which have or could be perceived to have influenced the work reported in this article.

## Appendix Supplementary materials

Image, application 1
